# Pharmacological inhibition of sphingolipid synthesis reduces ferroptosis by stimulating the HIF-1 pathway

**DOI:** 10.1016/j.isci.2022.104533

**Published:** 2022-06-06

**Authors:** Yang Liu, Libo He, Binghua Liu, Yuling Ying, Junling Xu, Meng Yu, Jinye Dang, Ke Liu

**Affiliations:** 1 Key Laboratory of Bio-Resource and Eco-Environment of Ministry of Education, College of Life Sciences, Sichuan University, Chengdu 610065 Sichuan, P.R.China; 2 Laboratory of Molecular Biology, College of Medicine, Chengdu University, Chengdu 610106 Sichuan, P. R. China

**Keywords:** Biological sciences, Neuroscience, Cellular neuroscience, Cell biology

## Abstract

Ferroptosis is crucial to the pathology of many neurological diseases. Here, we found pre-treatment with myriocin, an inhibitor of *de novo* synthesis of sphingolipid, significantly decreased the erastin- or glutamate-induced ferroptosis of HT22 cells without requiring the recovery of intracellular glutathione. The transcriptome analysis of HT22 cells treated with or without myriocin identified the hypoxia-inducible factor 1 (HIF-1) pathway as a prime and novel drug target. Further study validated that HIF1α was required for the cytoprotective effects of myriocin. Myriocin treatment promoted the expression of HIF-1 pathway effectors including PDK1 and BNIP3 and altered the intracellular levels of glucose metabolites. Additionally, myriocin treatment stabilized HIF1α protein by decreasing its ubiquitination and proteasomal degradation. Similar effects of myriocin on HIF1α stabilization were also found in other mammalian cell lines indicating this is a common mechanism for the cytoprotective role of myriocin.

## Highlights

• Inhibiting sphingolipid synthesis by myriocin reduces the ferroptosis
• Myriocin activates the HIF-1 pathway
• The effects of myriocin on ferroptosis require HIF1α
• Myriocin stabilizes HIF1α protein by reducing its ubiquitination

## Graphical abstract

**Figure:**
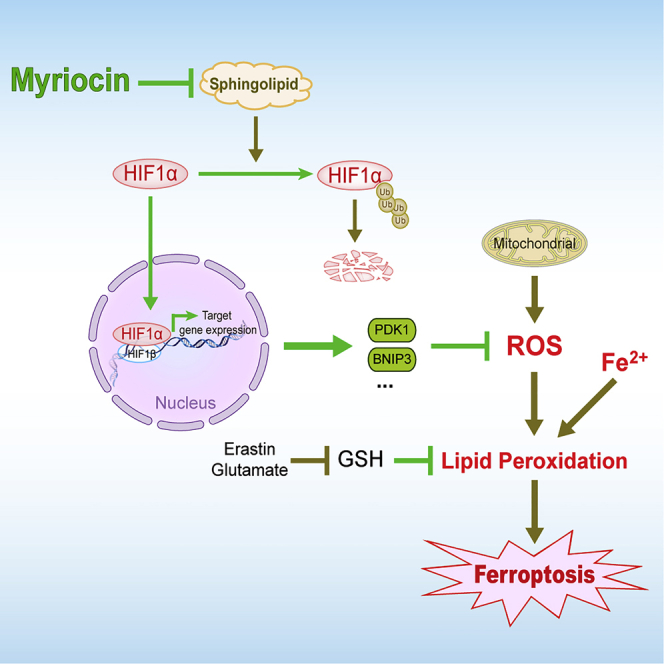

## Introduction

Oxytosis is a form of regulated cell death (RCD) first described in nerve cells and recently recognized as ferroptosis, which is characterized by iron-dependent, lipid peroxidation, and non-apoptotic cell death (Maher et al., 2020). Ferroptosis has been shown to have significant implications in many neurologic disorders, including stroke (Alim et al., 2019), Alzheimer disease (AD) (Bao et al., 2021), Parkinson disease (PD) (Mahoney-Sanchez et al., 2021), Huntington disease (Mi et al., 2019), and multiple sclerosis (MS) (Hu et al., 2019). While enhanced ferroptosis of cancer cells may benefit patients with cancer, reduced ferroptosis is desirable for the therapies of these neurological diseases. Ferroptosis in neuronal cells can be induced by the glutamate/cystine antiporter, system x_c_^−^, inhibitors (erastin or glutamate) (Jiang et al., 2020; Zhang et al., 2020). The inhibition of system x_c_^−^ results in glutathione depletion, excessive reactive oxygen species (ROS) production, and subsequent ferroptosis. It is important to note that in HT22 cells, N-methyl-*d*-aspartic-acid receptors (glutamate-gated cation channel) are absent and therefore this cell line is widely used to study glutamate-induced ferroptosis in neuronal cells (Maher and Davis, 1996). Although the *in vivo* relevance of system x_c_^−^ inhibition to neurologic disorders is not clear, the *in vitro* model of system x_c_^−^ inhibition has been extensively used for screening neuroprotective compounds targeting ferroptosis (Albrecht et al., 2010; Dixon et al., 2014).

Sphingolipids are important building blocks of cellular membranes and play key roles in the maintenance of cellular structural integrity. Additionally, sphingolipids, such as dihydrosphingosine (DHS), ceramides, and sphingosine-1-phosphate, regulate many cellular processes, including proliferation (Samarani et al., 2018), immunity (Seo et al., 2011), aging (Huang et al., 2012), auto/mitophagy (Bekhite et al., 2021), and apoptosis (Giussani et al., 2014). The *de novo* synthesis of sphingolipid is inhibited by myriocin, which specifically targets serine palmitoyl transferase (SPT) (Wadsworth et al., 2013). Myriocin, also called ISP-1, is a metabolite of the fungus *Isaria sinclairii* (Miyake et al., 1995). It resembles the transient intermediate formed by SPT in the condensation reaction of L-serine and palmitoyl-CoA (Chen et al., 1999). Our previous studies revealed that myriocin extends the lifespan of yeast cells by enhancing oxidative stress response and regulating homeostasis of intracellular amino acids (Hepowit et al., 2021; Liu et al., 2013). Recent studies have reported that limiting sphingolipid biosynthesis by myriocin had protective effects on many neurological diseases, including AD (Petit et al., 2020), PD (Lin et al., 2018), MS (Dasgupta and Ray, 2019), and Friedreich ataxia (Chen et al., 2016). However, the neuroprotective effect of myriocin against ferroptosis has not been addressed. In the present study, we investigated the neuroprotective effect of myriocin on erastin or glutamate-induced ferroptosis in HT22 cells and discovered that the HIF-1 pathway was stimulated by myriocin to attenuate ferroptosis.

## Results

### Myriocin protects HT22 cells against erastin- or glutamate-induced cell death

**Figure 1:**
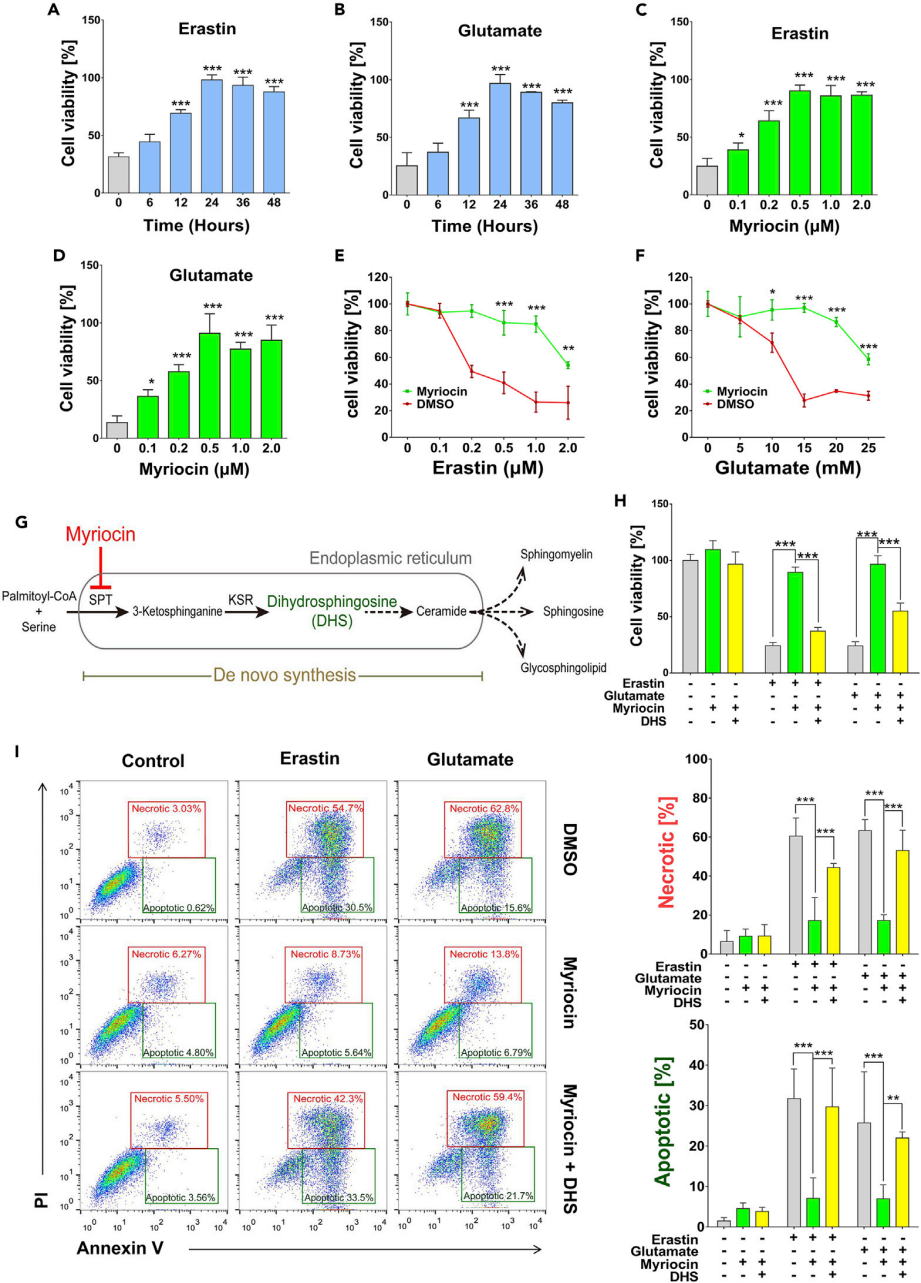
Myriocin inhibits erastin- or glutamate-induced ferroptosis in HT22 cells (A and B) Cell viability analysis of cells pre-treated with myriocin (0.5 μM) for indicated time before incubating with erastin (1 μM) (A) or glutamate (15 mM) (B) for 24 h. (C and D) Cell viability analysis of cells pre-treated with indicated concentrations of myriocin for 36 h before incubation with erastin (1 μM) (C) or glutamate (15 mM) (D) for 24 h. (E and F) Cell viability analysis of cells pre-treated with or without myriocin (0.5 μM) for 36 h before incubating with indicated concentrations of erastin (E) or glutamate (F) for 24 h. (G) Schematic diagram of the *de novo* biosynthesis pathway of sphingolipids in the ER SPT (serine palmitoyl transferase), and KSR (3-ketosphinganine reductase). (H) Cell viability analysis of cells pre-treated with or without myriocin (0.5 μM) or DHS (1 μM) for 36 h before incubating with or without erastin (1 μM) or glutamate (15 mM) for 24 h. (I) Annexin V-FITC/PI flow cytometric analysis of cells pre-treated with or without myriocin (0.5 μM) or DHS (1 μM) for 36 h before incubating with or without erastin (1 μM) or glutamate (15 mM) for 24 h. Histograms show numbers of necrotic and apoptotic cells. For the above, error bars represent the mean ± SD (n = 3, ∗p < 0.05, ∗∗p < 0.01, ∗∗∗p < 0.001).

The inhibition of system x_c_^−^ and the resulted oxidative damages of HT22 hippocampal neuronal cells by erastin- or glutamate-induced ferroptosis, which has been implied in the pathology of many neuronal diseases (Bueno et al., 2020; Chu et al., 2020; Neitemeier et al., 2017). To evaluate the potential neuronal cytoprotective effects of myriocin, HT22 cells were pre-treated with 0.5 μM of myriocin for different time from 6 to 48 h followed by treatment with erastin (1 μM) or glutamate (15 mM) for 24 h. The viability of HT22 cells without the pre-treatment of myriocin was significantly decreased by erastin or glutamate, while myriocin pre-treatment for 12 h or more provided significant protection (Figures 1A and 1B). The protective effect of myriocin is also concentration-dependent from 0.1 to 0.5 μM and the concentrations higher than 0.5 μM are not more beneficial (Figures 1C and 1D). Additionally, myriocin was able to protect HT22 cells against various concentrations of erastin or glutamate (Figures 1E and 1F). These data demonstrate that myriocin is an effective compound against erastin- or glutamate-induced death of HT22 cells.

Myriocin is a potent suicide inhibitor of SPT, the first and rate-determining enzyme which produces 3-ketosphinganine in the pathway of the *de novo* biosynthesis of sphingolipids (Wadsworth et al., 2013). 3-ketosphinganine is then converted to dihydrosphingosine (DHS) by 3-ketosphinganine reductase to produce ceramides and complex sphingolipids (Figure 1G). In order to validate the hypothesis that myriocin protects HT22 cells against ferroptosis by inhibiting the *de novo* biosynthesis of sphingolipids, the protective effect of myriocin on the viability of HT22 cells treated with erastin or glutamate was monitored in the presence of DHS. Indeed, the replenishment of DHS reduced the effect of myriocin on cell viability when DHS alone had no impact on cell viability (Figures 1H and S1). The flow cytometric analysis of dying cells revealed that after 24 h of glutamate or erastin exposure, a major portion of cells was FITC Annexin V positive and PI positive (necrotic or ferroptotic) and a relatively smaller portion of cells was FITC Annexin V positive and PI negative (apoptotic) (Figure 1I). The pre-treatment of myriocin significantly mitigated the appearance of either apoptotic or necrotic cells, suggesting that myriocin may protect cells from both ferroptosis and apoptosis. And the co-treatment of DHS with myriocin reversed the protective effect of myriocin (Figure 1I). Therefore, the protective effect of myriocin on cell viability relies on its inhibition of DHS production.

### Myriocin decreases erastin- or glutamate-induced oxidative damage independent of GSH

**Figure 2:**
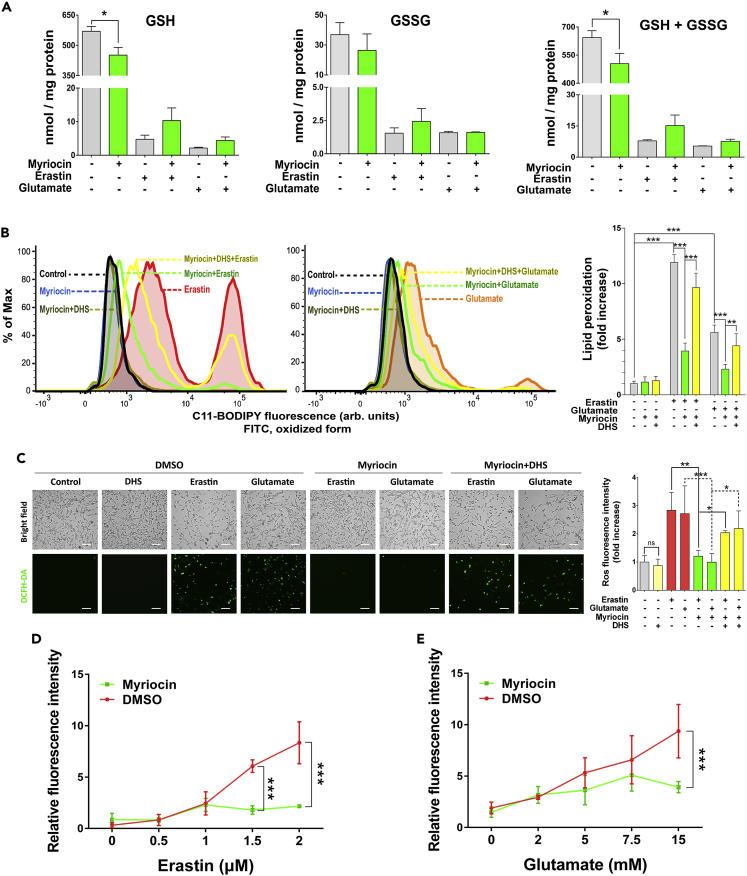
Myriocin inhibits oxidative damage induced by erastin or glutamate treatment HT22 cells were pre-treated with or without myriocin (0.5 μM) or DHS (1 μM) for 36 h before incubating with or without erastin (1 μM) or glutamate (15 mM) for 24 h. (A) Intracellular glutathione levels of cells treated as indicated. (B) Lipid peroxidation of cells treated as indicated and monitored by flow cytometry after labeling with C11-BODIPY 581/591, fluorescence intensity was measured on the FITC channel (left two panels: distributions of fluorescence, right panel: relative quantification of fluorescence enhancement which represents the extent of probe oxidation). (C) Intracellular ROS production of cells treated as indicated and visualized by using the fluorescent probe DCFH-DA, scale bar: 50 μm. Bar graph shows the quantification of fluorescence intensity relative to the relevant untreated control. (D and E) Relative level of labile iron pool (LIP) in cells treated as indicated and monitored by the calcein fluorescence quenching method. For the above, error bars represent the mean ± SD (n = 3, ∗p < 0.05, ∗∗p < 0.01, ∗∗∗p < 0.001).

Erastin- and glutamate-induced ferroptosis in neuronal cells is initiated by the inhibition of the Na^+^-independent cystine/glutamate antiporter, system x_c_^−^, which in turn prevents the uptake of cystine and blocks the synthesis of glutathione (Murphy et al., 1989). To investigate if the cytoprotective role of myriocin against erastin- or glutamate-induced cell death is related to the homeostasis of glutathione, we monitored the effect of myriocin on the levels of oxidative and reductive glutathione in HT22 cells. Erastin or glutamate treatment greatly decreased the level of both reduced and oxidized glutathione as expected from previous studies (Figure 2A) (Herrera et al., 2007; Wang et al., 2020). The pre-treatment of myriocin alone slightly decreased the level of glutathione in cells before erastin or glutamate treatment and it did not recover glutathione levels after erastin or glutamate treatment (Figure 2A). Therefore, the cytoprotective role of myriocin does not rely on the restoration of GSH level.

Because most of dying cells are ferroptotic (Figure 1I), we sought to understand how myriocin attenuates erastin- and glutamate-induced ferroptosis. We monitored lipid peroxidation, a hallmark of ferroptosis, and found that while erastin or glutamate treatment significantly enhanced lipid peroxidation 12-fold or 6-fold, respectively, pre-treatment with myriocin significantly suppressed these effects as revealed by flow cytometry using the fluorescent probe C11-BODIPY (Figure 2B). The effect of myriocin on lipid peroxidation was also inhibited by DHS (Figure 2B). Additionally, the production of ROS in HT22 cells was analyzed by using a fluorescent probe DCFH-DA. While the pre-treatment of myriocin abolished the surge of ROS production induced by erastin or glutamate treatment, the addition of DHS counteracted the effects of myriocin, and DHS alone had no impact on ROS production (Figure 2C). Because ferroptosis is iron-dependent, the relative labile iron pool (LIP) level in HT22 cells was monitored by the calcein fluorescence quenching method. Both erastin and glutamate treatments increased intracellular LIP in a concentration-dependent manner and the pre-treatment of myriocin significantly attenuated the increase of intracellular LIP induced by erastin or glutamate treatment (Figures 2D and 2E). These data suggest that inhibiting the biosynthesis of sphingolipids by myriocin protected HT22 cells against erastin- or glutamate-induced ferroptosis by suppressing oxidative damage.

### Myriocin activates the HIF-1 signaling pathway

**Figure 3:**
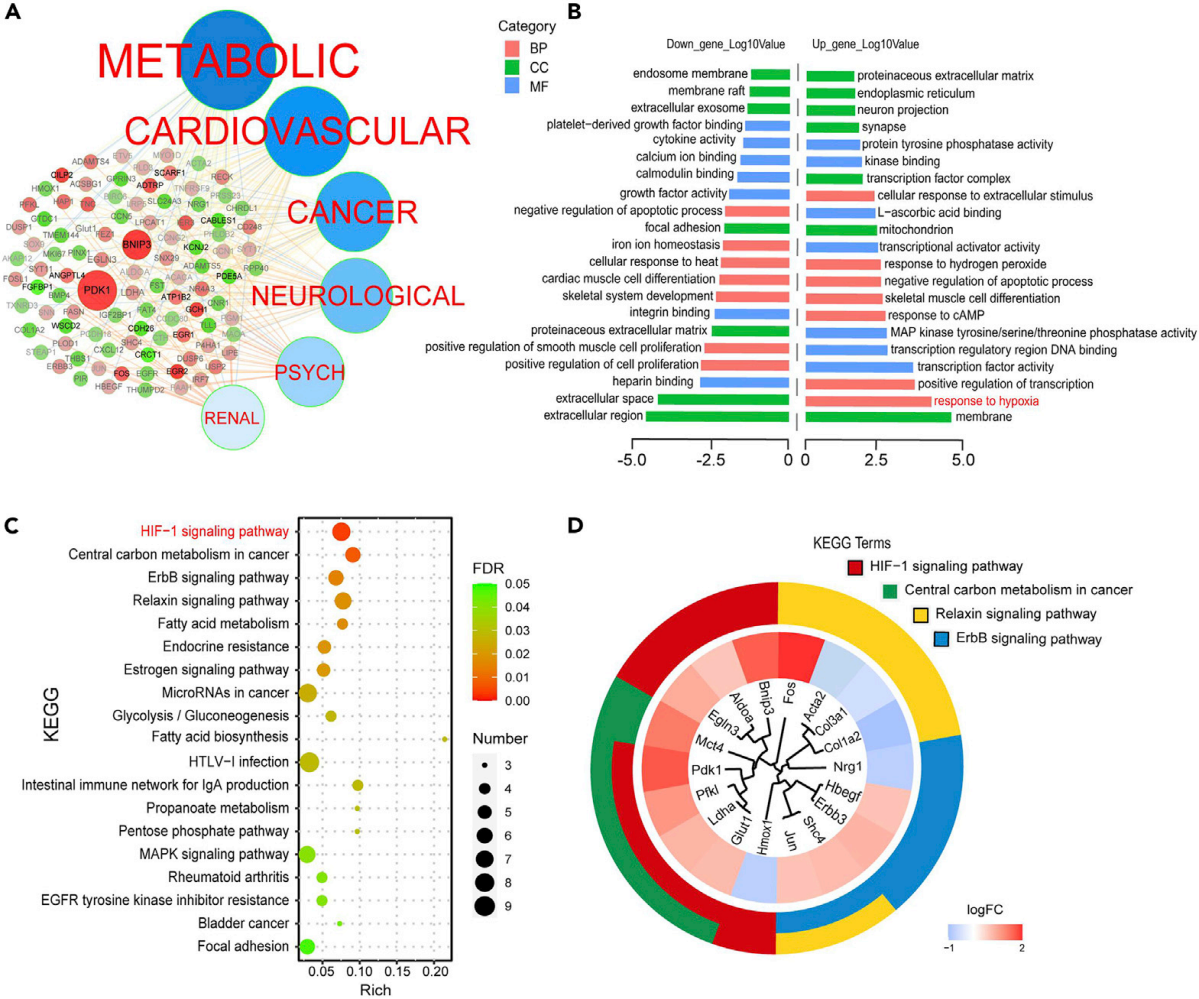
Transcriptome profiles of HT22 cells treated with myriocin HT22 cells treated with myriocin (0.5 μM) for 36 h were analyzed for differential expression genes (DEGs, n = 3, p < 0.05). (A) Disease and DEGs correlation network. The size of the knots represents the p value of DEGs, and the red and green color represent the up- and downregulated genes, respectively. (B) Gene Ontology (GO) annotation for up- and downregulated DEGs (BP, biological process, CC, cell component, MF, molecular function). (C) KEGG pathway enrichment analysis of up- and downregulated DEGs. Numbers of enriched genes, enrichment scores, and FDR are presented. (D) Circular dendrogram of the top four KEGG clusters.

To gain insight into the cytoprotective mechanism of myriocin in HT22 cells, transcriptome profiles of HT22 cells treated with or without 0.5 μM of myriocin were investigated and compared. We identified 113 upregulated and 71 downregulated differentially expressed genes (DEGs) upon myriocin treatment (Figure S2 and Table S1). Disease and gene correlation network established by homology analysis of DEGs with *Homo sapiens* genome demonstrates that myriocin treatment regulated the expression of genes involved in metabolic diseases, cardiovascular diseases, cancer, and neurological diseases (Figure 3A). Gene Ontology (GO) analysis indicates that the downregulated genes were mainly related to cell proliferation and extracellular regulation (Figure 3B, left part). For upregulated genes, top three GO terms in biological process (BP) enrichment were “response to hypoxia”, “positive regulation transcription”, and “response to cAMP”. The top three GO terms in cell component (CC) enrichment were “membrane”, “mitochondrion”, and “transcription factor complex”. And the top three GO terms in molecular function (MF) enrichment were “transcription factor activity”, “transcription regulatory region DNA binding”, and “MAP kinase activity” (Figure 3B, right part). These results suggest that myriocin treatment activated the transcription factors related to hypoxia. Additionally, Kyoto Encyclopedia of Genes and Genomes (KEGG) analysis of the DEGs identified 19 signaling pathways significantly altered by myriocin treatment (Figure 3C). Among them, HIF-1 signaling pathway was the term with the lowest false discovery rate (FDR) second by the term of central carbon metabolism in cancer (Figure 3C). Note that there was an apparent overlapping of DEGs between these two terms (Figure 3D). These transcriptome data strongly suggest that myriocin may activate the HIF-1 signaling pathway.

**Figure 4:**
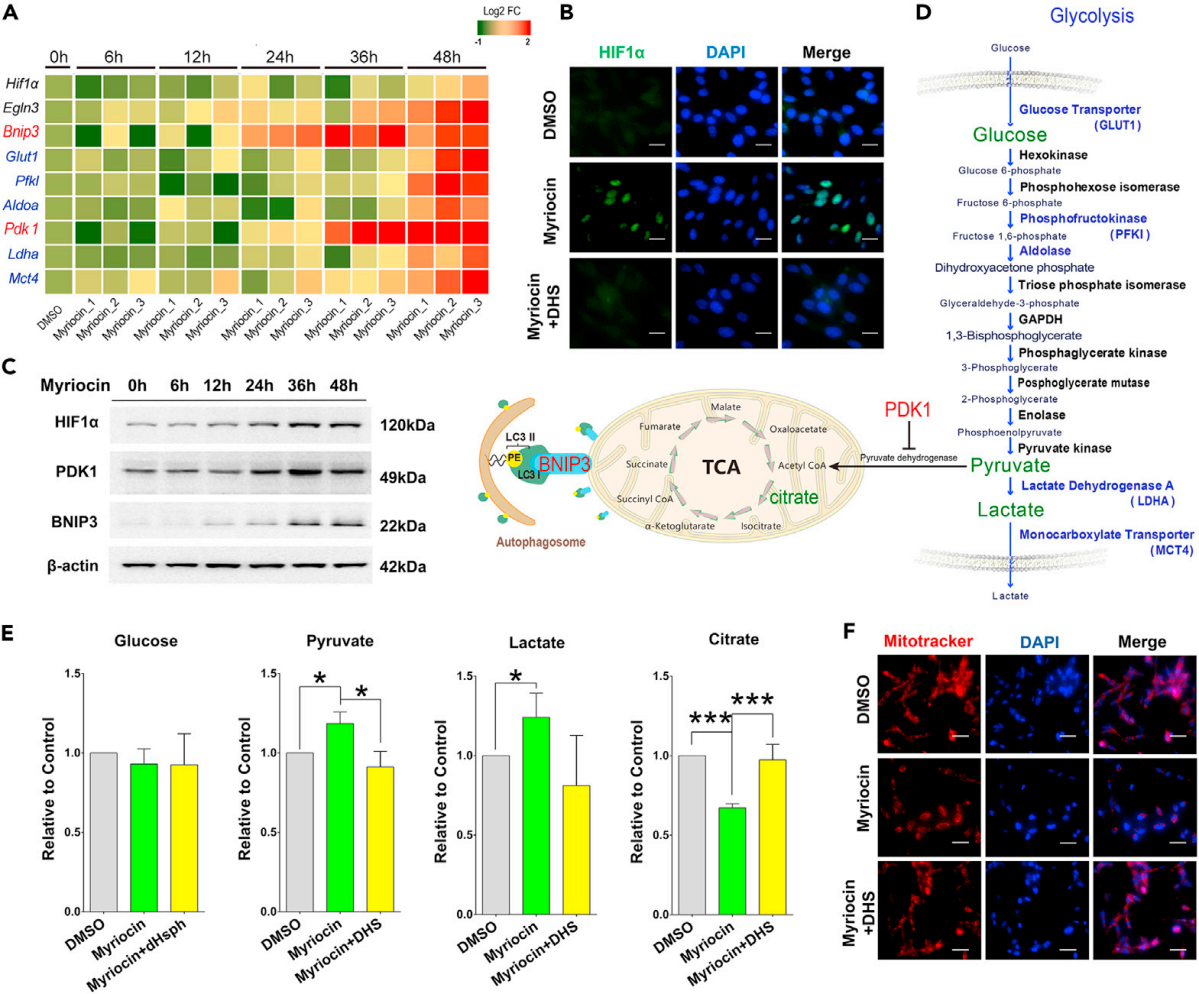
Myriocin activates the HIF-1 signaling pathway (A) HT22 cells treated with myriocin (0.5 μM) for indicated time were subjected to qPCR to measure the relative mRNA expression of indicated genes. Heatmap showing the log2 fold change (FC) between myriocin treatment and control samples of triplicate experiments. (B) HT22 cells were treated with myriocin (0.5 μM) in the presence or absence of DHS (1 μM) for 36 h. HIF1α (green) was monitored by immunofluorescence staining and nuclei were stained with DAPI (blue), scale bar: 20 μm. (C) HT22 cells treated with myriocin (0.5 μM) for indicated time were subjected to Western blotting to monitor the protein levels of HIF1α, PDK1, and BNIP3. (D) Schematic diagram of glycolysis, tricarboxylic acid (TCA) cycle, and mitophagy which are regulated by PDK1 and BNIP3. Glucose and its metabolites including pyruvate, lactate, and citrate which were monitored in this study are marked in green. During glycolysis, one molecule of glucose yields two molecules of pyruvate. Pyruvate is converted to Acetyl CoA by pyruvate dehydrogenase and enters the TCA cycle. The activity of pyruvate dehydrogenase can be inhibited by the phosphorylation induced by PDK1. BNIP3 accumulated on the outer mitochondrial membrane may interact with LC3 to promote mitophagy. (E) Relative levels of intracellular glucose, pyruvate, lactate, and citrate in HT22 cells after treatment with or without myriocin (0.5 μM) or DHS (1 μM) for 36 h. Error bars represent the mean ± SD (n = 3, ∗p < 0.05, ∗∗p < 0.01, ∗∗∗p < 0.001). (F) With or without myriocin (0.5 μM) or DHS (1 μM) treatment for 36 h, mitochondria and nuclei of HT22 cells were detected by fluorescence microscopy after labeling with MitoTracker (red) and DAPI (blue).

To validate the DEGs regulated by HIF-1 signaling pathway, the expression levels of *Eglh3*, *Bnip3*, *Glut1*, *Pfkl, Aldoa, Pdk1, Ldha,* and *Mct4* were analyzed by qPCR in HT22 cells treated from 6 to 48 h with myriocin. All of these transcripts were increased by myriocin treatment (Figure 4A). Note that there was only a very minor increase in the mRNA level of *Hif1α* which is the central regulator of HIF-1 signaling pathway, consistent with the transcriptome analysis (Table S1). However, the immunofluorescence images indicate that the protein level of HIF1α was remarkably elevated by myriocin treatment (Figure 4B), suggesting that it was regulated post-transcriptionally by myriocin. Western blots also reveal that the level of HIF1α protein, together with PDK1 and BNIP3 proteins were significantly increased over the time in myriocin-treated cells (Figures 4C and S3). Furthermore, this increase in protein levels was prevented by supplementing DHS (Figures 4B and S4).

PDK1 and BNIP3 are among the mostly studied proteins controlled by HIF-1 and play crucial roles in regulating the catabolism of glucose and mitochondrial autophagy, respectively (Figure 4D) (Kim et al., 2006; Zhang et al., 2008). Therefore, we first asked if the catabolism of glucose was altered by myriocin. Indeed, myriocin treatment inhibited the carbon flux from glycolysis to tricarboxylic acid (TCA) cycle in HT22 cells as revealed by the elevation of intracellular levels of pyruvate and lactate and the reduction of citrate when intracellular glucose was constant (Figure 4E). This phenomenon resembled the effects of PDK1 upregulation. Additionally, myriocin treatment decreased the content of mitochondrial (Figure 4F) and increased LC3II, a marker of autophagy (Figure S5). These changes are consistent to the effects of BNIP3 upregulation. These effects of myriocin were also inhibited by the addition of DHS (Figures 4E and 4F). Together, these data indicate that myriocin induced the activation of HIF1α and its downstream signals by inhibiting the *de novo* synthesis of sphingolipids.

### The cytoprotective effects of myriocin require HIF1α

**Figure 5:**
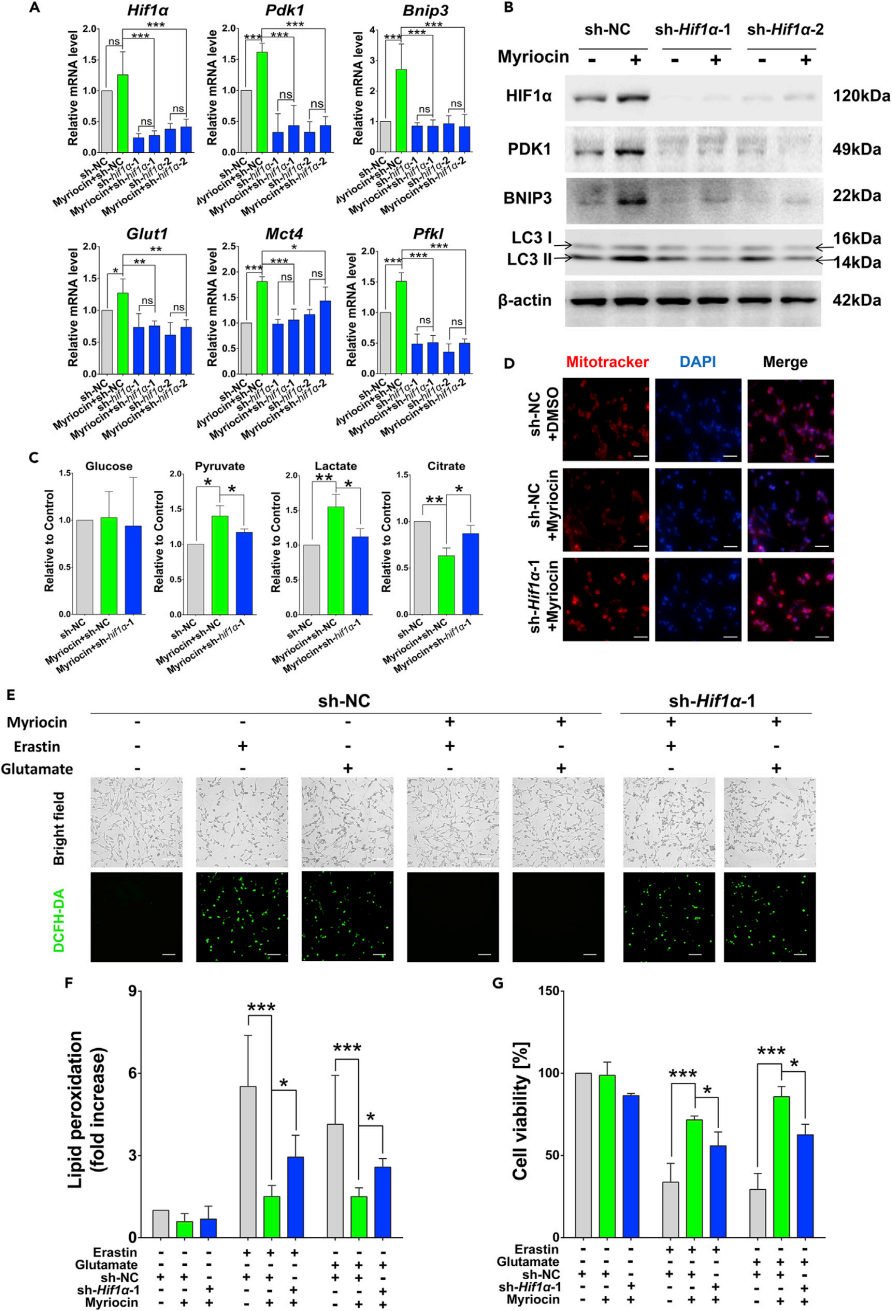
Cytoprotective effects of myriocin requires HIF1α (A) qPCR analysis for the relative expression of indicated genes in HT22 cells transfected with small hairpin RNA (sh-*Hif1α*-1, sh-*Hif1α*-2) or non-targeting sequence (sh-NC) after treatment with or without myriocin (0.5 μM) for 36 h. (B) Proteins of interest in HT22 cells transfected with sh-*Hif1α*-1, sh-*Hif1α*-2, or sh-NC were tested by Western blotting after treatment with or without myriocin (0.5 μM) for 36 h. (C) Relative levels of intracellular glucose, pyruvate, lactate, and citrate in HT22 cells transfected with sh-*Hif1α*-1 or sh-NC after treatment with or without myriocin (0.5 μM) for 36 h. (D) With or without myriocin (0.5 μM) treatment for 36 h, mitochondria and nuclei in HT22 cells with indicated treatments were stained by MitoTracker (red) and DAPI (blue) before imaged by fluorescence microscopy, scale bar: 20 μm. (E) Treatment with or without myriocin (0.5 μM) for 36 h before incubating with or without erastin (1 μM) or glutamate (15 mM) for 24 h, the intracellular ROS of HT22 cells was detected by fluorescence microscopy after labeling with DCFH-DA probe, scale bar: 50 μm. (F) Flow cytometry analysis of HT22 cells with indicated treatments (E) after labeled with C11-BODIPY 581/591. Relative fold changes of lipid peroxidation were calculated by quantifying oxidized probe. (G) Cell viability of HT22 cells after indicated treatments (E) was analyzed by CCK-8 method. For the above, error bars represent the mean ± SD (n = 3, ∗p < 0.05, ∗∗p < 0.01, ∗∗∗p < 0.001).

To investigate the role of HIF1α in the cytoprotective effects of myriocin, *Hif1α* was knocked down in HT22 cells. *Hif1α* knockdown resulted in a drastic decrease of its mRNA and protein levels (Figures 5A, 5B, and S6), and suppressed the expression of HIF-1 effectors including *Pdk1*, *Bnip3*, *Glut1*, *Mct4*, and *Pfkl* which were stimulated by myriocin treatment (Figure 5A). Additionally, the expression of PDK1 and the intracellular levels of glucose, pyruvate, lactate, and citrate did not respond to myriocin treatment anymore after *Hif1α* knockdown (Figures 5A, 5B, 5C and S6), indicating that the regulation of PDK1 and glycolysis by myriocin requires HIF1α. Similarly, consistent with the expression of *Bnip3* (Figures 5A, 5B, and S6), the mitochondrial content of cells after *Hif1α* knockdown was not decreased by myriocin treatment (Figure 5D). Additionally, *Hif1α* knockdown hampered the inhibitory effect of myriocin on ROS production and lipid peroxidation induced by erastin or glutamate treatment (Figures 5E and 5F). Lastly, the protective effect of myriocin against erastin- or glutamate-induced cell death was significantly reduced by *Hif1α* knockdown Figure 5G). These results strongly indicate that HIF1α is crucial for the cytoprotective effects of myriocin against erastin or glutamate treatment in HT22 cells.

### Myriocin treatment stabilizes HIF1α by decreasing its ubiquitination

**Figure 6:**
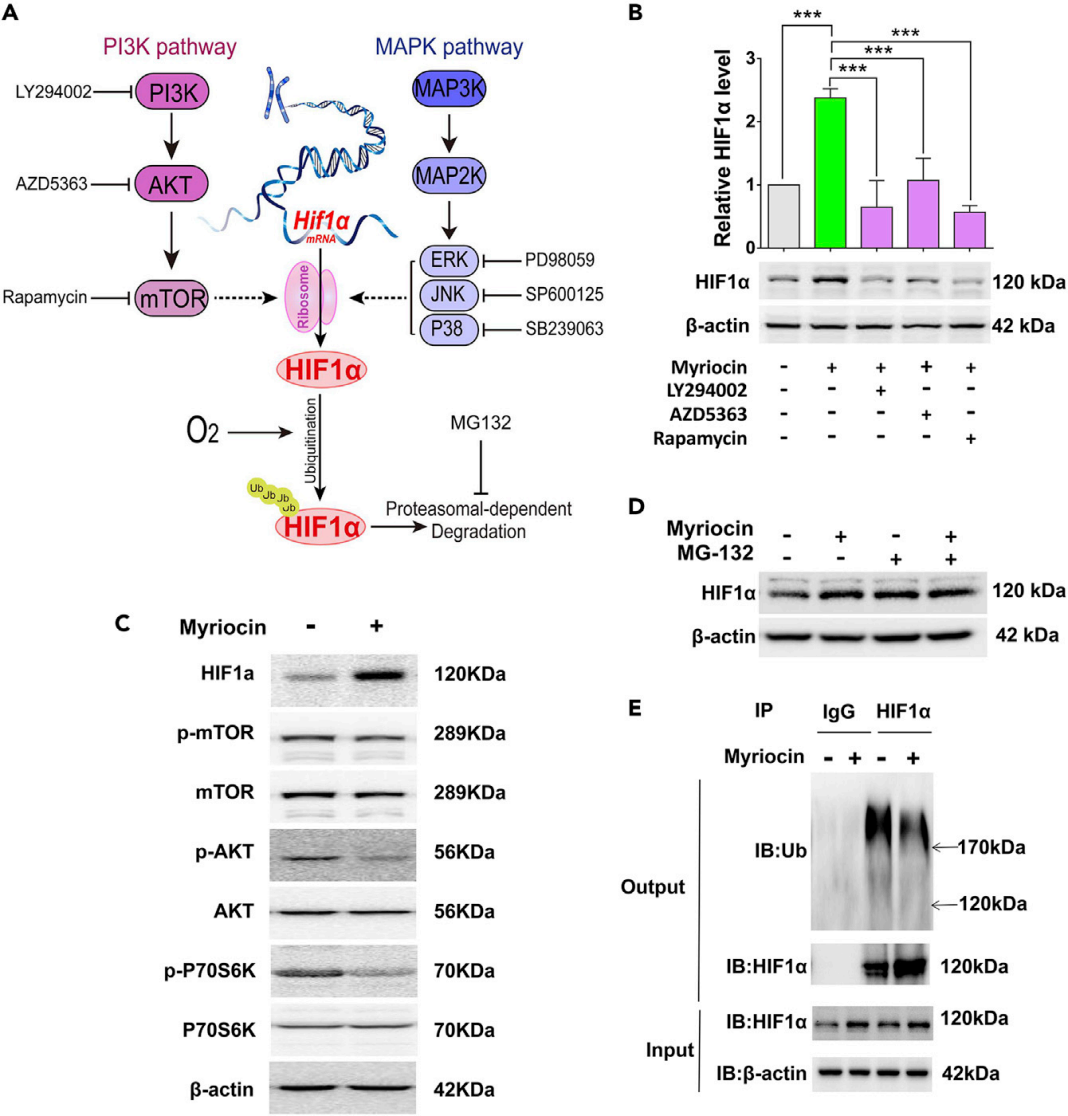
Myriocin treatment decreases ubiquitination of HIF1α (A) Diagram of HIF1α expression regulated post-transcriptionally during normoxia. Stimulation of the PI3K or MAPK signaling pathways increases the level of HIF1α by enhancing its translation. Ubiquitination of HIF1α promotes its degradation by proteasomes. (B) Western blotting analysis of HIF1α protein in HT22 cells treated with myriocin (0.5 μM) in the presence or absence of LY294002 (10 μM), AZD5363 (5 μM), or rapamycin (1 μM) for 36 h. Error bars represent the mean ± SD (n = 3, ∗∗∗p < 0.001). (C) Proteins of interest were analyzed by Western blotting after HT22 cells were treated with or without myriocin (0.5 μM). (D) Western blotting analysis of HIF1α protein in HT22 cells treated with or without myriocin (0.5 μM) in the presence or absence of MG132 (10 nM, 36 h). (E) Ubiquitin/HIF1α co-immunoprecipitation in HT22 cell extracts. Cells treated with or without myriocin (0.5 μM) were lysed and immunoprecipitated with anti-HIF1α antibody or normal immunoglobulin G (IgG) as negative control. The immunocomplexes were then immunoblotted using anti-ubiquitin or anti-HIF1α antibody (Output). The levels of HIF1α in cell lysates before immunoprecipitation were also monitored (Input).

HIF1α undergoes rapid degradation in normoxia, and its accumulation is controlled by the rates of both synthesis and degradation. Our results demonstrate that myriocin significantly stimulates the protein level of HIF1α with only very minor effect in the level of *Hif1α* mRNA (Figures 4A–4C), indicating that myriocin regulates HIF1α level mainly via post-transcriptional events. It has been suggested that the MAPK and PI3K pathways may promote translation of HIF1α (Figure 6A) (Befani et al., 2012; Masoud and Li, 2015; Mi et al., 2017). We found that inhibiting the MAPK pathway did not interfere with HIF1α expression in HT22 cells (Figure S7A). On the other hand, when HT22 cells were treated with LY294002, AZD5363, or rapamycin, inhibitors of PI3K, AKT, and mTOR, respectively, HIF1α was significantly decreased even when cells were co-treated with myriocin (Figures 6B and S7B). However, myriocin treatment did not increase, but instead decreased the phosphorylation of AKT and P70S6K (Figures 6C and S7C), indicating that myriocin inhibits the PI3K/AKT/mTOR axis. Therefore, although the level of HIF1α is indeed regulated by PI3K/AKT/mTOR axis, myriocin did not increase HIF1α by activating this pathway.

Because HIF1α is degraded by proteasome, a proteasome inhibitor MG-132 was added into cell culture with or without myriocin treatment. The co-treatment of both MG-132 and myriocin did not further increase the HIF1α level which was elevated by MG-132 or myriocin alone (Figures 6D and S7D), indicating that there are some overlapping effects between myriocin and MG132. A cycloheximide chase assay also revealed that HIF1α protein is much more stable in cells pre-treated with myriocin (Figure S7E). These data indicate that myriocin may stabilize HIF1α by preventing its proteasomal degradation.

To investigate if the ubiquitination of HIF1α is regulated by myriocin, HIF1α protein was immunoprecipitated and its ubiquitination level was analyzed. While myriocin treatment increased the level of the un-ubiquitinated form of HIF1α, ubiquitinated HIF1α was decreased to about 50% (Figures 6E and S7F). And the addition of DHS counteracted this effect (Figure S7G). Together, these results suggest that myriocin inhibits the proteasomal degradation of HIF1α by partially preventing its ubiquitination.

**Figure 7:**
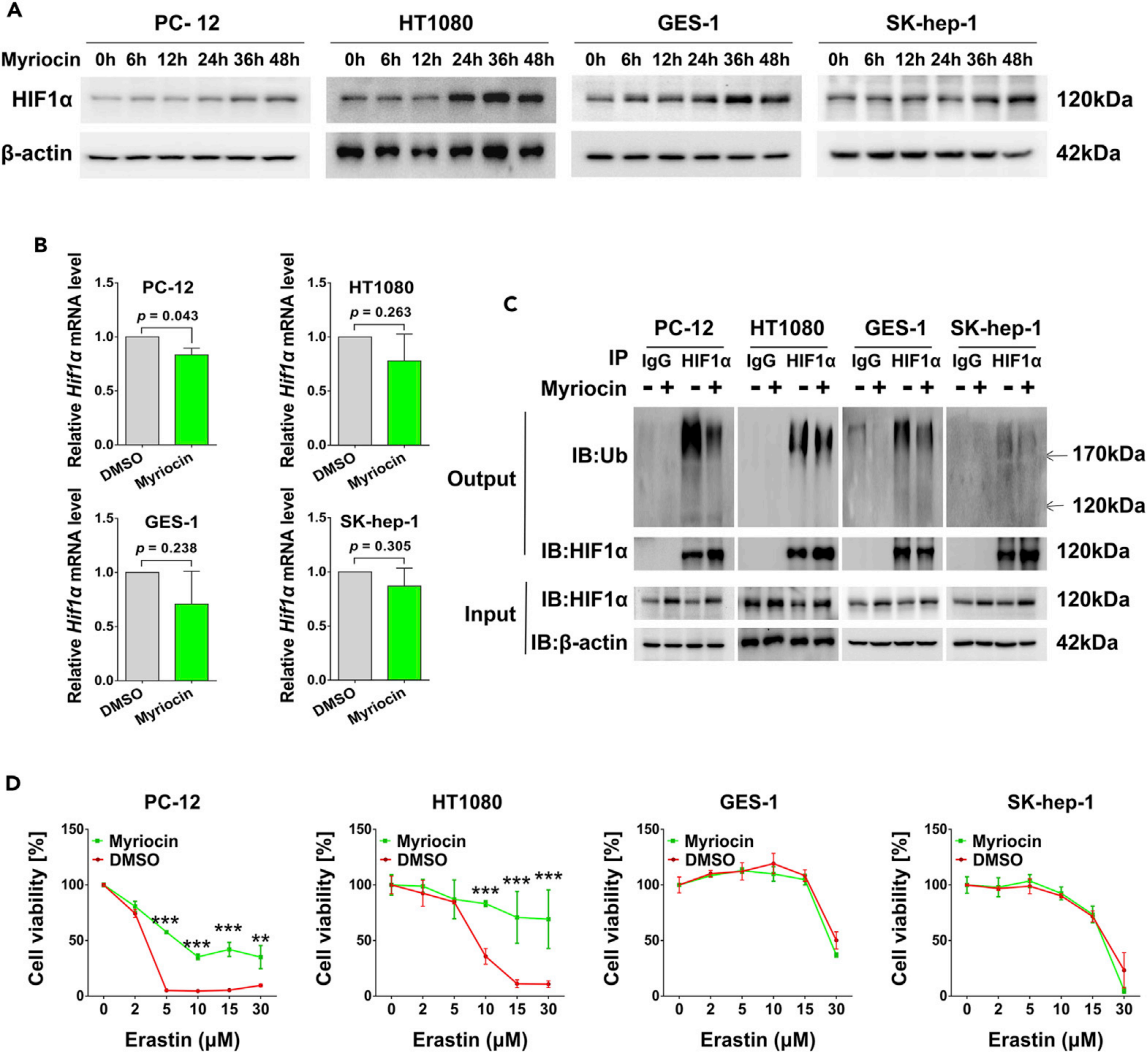
Cytoprotective effects of myriocin in different cells (A) PC-12, HT1080, GES-1, and SK-Hep-1 cells treated with myriocin (0.5 μM) for indicated time were subjected to Western blotting to monitor the protein levels of HIF1α. (B) Cells treated with myriocin (0.5 μM) for 36 h were subjected to qPCR to measure the relative Hif1α mRNA expression. Error bars represent the mean ± SD (n = 3). (C) Ubiquitin/HIF1α co-immunoprecipitation in cells extracts. Cells treated with or without myriocin (0.5 μM) were lysed and immunoprecipitated with anti-HIF1α antibody or IgG as negative control. The immunocomplexes were then immunoblotted using anti-ubiquitin or anti-HIF1α antibody (Output). The levels of HIF1α in cell lysates before immunoprecipitation were also monitored (Input). (D) Cell viability analysis of cells pre-treated with myriocin (0.5 μM) for 36 h before incubating with erastin for 24 h. Error bars represent the mean ± SD (n = 3, ∗∗p < 0.01, ∗∗∗p < 0.001).

Because HIF-1 pathway plays important roles in many physiological and pathological processes in all metazoan organisms, we asked if myriocin also upregulates HIF1α in cells other than HT22. Therefore, the levels of HIF1α in four other cell lines including PC-12 (rat neural cell), HT1080 (human fibrosarcoma cell), GES-1 (human gastric epithelial cell), and SK-Hep-1 (human hepatoma cell) were monitored with or without myriocin treatment. Myriocin treatment increased the protein level of HIF1α in all of these cell types (Figures 7A and S8A) with no effect on the mRNA levels (Figure 7B). Additionally, the ubiquitination of HIF1α in PC-12, HT1080, and GES-1 cells was inhibited by myriocin (Figures 7C and S8B). The baseline ubiquitination of HIF1α in SK-Hep-1 was too low to observe a further decrease (Figures 7C and S8B). Pre-treatment with myriocin decreased the erastin-induced cell death of PC-12 and HT1080 cells, but it did not promote the survival of GES-1 and SK-Hep-1 cells which had higher basal levels of HIF1α than PC-12, HT1080, and HT22 cells (Figures 7D and S8C). Therefore, the effect of myriocin on HIF1α is not limited in HT22 although its effects on cell fate are cell-type-dependent.

## Discussion

It has been demonstrated that inhibiting *de novo* synthesis of sphingolipids by myriocin induces stress responses and increases lifespan in yeast cells. This cytoprotective effect of myriocin seems to be conserved in eukaryotes because myriocin treatment also increases lifespan in *Caenorhabditis elegans* (Cutler et al., 2014). In rodent models, myriocin treatment mitigates age-related diseases including Alzheimer disease, motor neuron degeneration, and Parkinson disease (Geekiyanage et al., 2013; Lin et al., 2019; Petit et al., 2020). The cytoprotective effects of myriocin on yeast cells are established by inducing a state resembling amino acid restriction and the subsequent inhibition of mTOR pathway (Hepowit et al., 2021). However, it is unclear how inhibiting sphingolipid synthesis by myriocin promotes cell survival in metazoan organisms. To facilitate the therapeutic usage of myriocin or myriocin-like compounds, determining the mechanistic basis of the cytoprotective effect induced by myriocin in mammalian cells is critical. Here, we report that myriocin inhibits ferroptosis induced by glutathione depletion in neuronal HT22 cells. This is so far the first study demonstrating that inhibiting the synthesis of sphingolipids protects cells against ferroptosis. Because ferroptosis induced by oxidative stress in neuronal cells is relevant to aging and age-related neurodegeneration, our work provides a new approach for developing therapeutic or preventive means against those disorders.

Erastin or glutamate treatment in HT22 cells results in glutathione depletion which in turn induces ferroptosis. Glutathione is required for the removal of lipid peroxides by glutathione peroxidase 4 (GPx4), a key enzyme to protect cells from ferroptotic death under oxidative stress. The fact that myriocin treatment barely prevents the drop of intracellular levels of glutathione, suggests that the protective effect of myriocin do not rely on the scavenging of lipid peroxide by GPx4. Recent studies have revealed that lipid peroxides can be removed by FSP1 through ubiquinone (CoQ10) or by nitric oxide synthase (iNOS)/NO independent of GPx4 (Doll et al., 2019; Kapralov et al., 2020), which shares a similar mechanical character as the protective effects of myriocin. However, myriocin pre-treatment significantly suppressed ROS production under glutathione depletion. Additionally, the level of LIP was also decreased, most likely due to the less ROS which leads to the releasement of iron from its storage proteins (Di Tano et al., 2020; Lu and Imlay, 2019; Torti and Torti, 2013). Unlike GPx4 or FSP1, the protective role of myriocin against ferroptosis relies on the prevention of lipid peroxide accumulation caused by the oxidative reaction of polyunsaturated fatty acid with ROS and iron (Boada-Romero et al., 2020; Tang et al., 2019). Therefore, it is likely that the removal of lipid peroxides contributes very little, if any, to the protective effects of myriocin against ferroptosis.

To address how myriocin prevents the buildup of oxidative stress induced by glutathione depletion, we performed transcriptome analysis and surprisingly identified the HIF-1 pathway. Although it has been well established that this pathway is involved in cell survival and the inhibition of ferroptosis (Liu et al., 2019; Poloznikov et al., 2020; Yang et al., 2019), it has never been reported that inhibiting biosynthesis of sphingolipids activates HIF-1 pathway. Additionally, although some studies have demonstrated that HIF-1 pathway or hypoxia plays substantial roles in sphingolipid metabolism (Novgorodov and Gudz, 2011; Testai et al., 2014), if and how sphingolipid metabolism affects HIF-1 pathway is not well established. By validating the activation effect of myriocin on the HIF-1 pathway, this work identifies a novel and interesting connection between sphingolipid metabolism and the HIF-1 pathway. There are many downstream effectors in the HIF-1 pathway which impact many aspects of cell physiology. In this study, we examined PDK1 and BNIP3 because they are key regulators of glycolysis and mitochondrial homeostasis, respectively. Because glycolysis and mitochondrial homeostasis are crucial for energy metabolism and ROS production, their alteration by myriocin is likely to be cytoprotective.

Finally, the regulatory role of myriocin on the level of HIF1α is pinpointed to the proteolysis process because the regulation at transcriptional or translational level has been ruled out. The proteolysis of HIF-1α is initiated by the binding of the von Hippel-Lindau tumor-suppressor protein (VHL), which in turn favors the ubiquitination of HIF1α and targets it to proteasomal degradation (Paltoglou and Roberts, 2007). Although further studies are required to reveal the details about how myriocin alters this process, our results indicate that the ubiquitination of HIF1α is decreased by myriocin treatment which results in the upregulation of HIF1α.

In conclusion, our work demonstrates that inhibiting the *de novo* synthesis of sphingolipid by myriocin protects neuronal cells against ferroptosis via the HIF-1 pathway. Mechanistically, myriocin increases HIF1α protein by inhibiting its ubiquitination and subsequent proteasomal degradation. Elevated HIF1α in turn stimulates the HIF-1 pathway which promotes cell survival against ferroptosis by rewiring carbon metabolism and promoting redox balance. Future works will aim to understand the cytoprotective roles of myriocin on *in vivo* models of neurodegeneration to evaluate the potential of myriocin or similar compounds for the prevention or treatment of multiple age-associated neurological disorders.

### Limitations of the study

Our data revealed the link between sphingolipid, HIF-1 pathway, and ferroptosis, but the exact underlying mechanism remains to be explored, especially the relationship between sphingolipid synthesis and HIF1α ubiquitination. And *in vivo* experiments are needed to evaluate the systemic effect of myriocin.

## STAR★Methods

### Key resources table

**Table:** 

REAGENT or RESOURCE	SOURCE	IDENTIFIER
**Antibodies**		
Rabbit monoclonal anti-HIF1α	Cell Signaling Technology	Cat#36169,RRID:AB_2799095
Rabbit polyclonal anti-Bnip3	Cell Signaling Technology	Cat# 3769, RRID:AB_2259284
Mouse monoclonal anti-Ubiquitin	Cell Signaling Technology	Cat# 3936, RRID:AB_331292
Rabbit monoclonal anti-p70 S6 Kinase	Cell Signaling Technology	Cat# 2708, RRID:AB_390722
Rabbit monoclonal anti-p70 S6 Kinase, phospho (Thr389)	Cell Signaling Technology	Cat# 9234, RRID:AB_2269803
Rabbit monoclonal anti-AKT1/2/3	Abcam	Cat# ab179463, RRID:AB_2810977
Rabbit monoclonal anti-mTOR	Abcam	Cat# ab134903, RRID:AB_2800465
Rabbit monoclonal anti-mTOR (phospho S2448)	Abcam	Cat# ab109268, RRID:AB_10888105
Rabbit monoclonal anti-AKT1/2/3(phospho S472 + S473 + S474)	Abcam	Cat# ab192623
Rabbit polyclonal anti-LC3A/B	Zen BioScience	Cat# 306019
Mouse monoclonal anti-beta Actin	Zen BioScience	Cat# 700068
Rabbit polyclonal anti-α-Tubulin	Beyotime Biotechnology	Cat# AF0001
HRP-labeled Goat Anti-Mouse IgG(H+L)	Beyotime Biotechnology	Cat# A0216
HRP-labeled Goat Anti-Mouse IgG(H+L)	Beyotime Biotechnology	Cat# A0216
FITC-labeled Goat Anti-Rabbit IgG (H+L)	Beyotime Biotechnology	Cat# A0562
Bacterial and virus strains pLKO.1-puro shHif1α-1	This paper	N/A
pLKO.1-puro shHif1α-2	This paper	N/A

**Chemicals, peptides, and recombinant proteins**		
Rapamycin	APExBIO Technology LLC	Cat# A8167, CAS:53123-88-9
Glutamate	APExBIO Technology LLC	Cat# C6110, CAS: 56-86-0
Erastin	APExBIO Technology LLC	Cat# B1524, CAS: 571203-78-6
Myriocin	Cayman	Cat# 63150, CAS: 35891-70-4
dihydrosphingosine	Sigma	Cat# D3314, CAS: 764-22-7
MG132	APExBIO Technology LLC	Cat# A2585, CAS: 133407-82-6

### Resource availability

#### Lead contact

Further information and requests for resources and reagents should be directed to and will be fulfilled by the lead contact, Ke Liu (kliu@scu.edu.cn).

#### Materials availability

This study did not generate any unique materials.

### Experimental model and subject details

#### Cell culture

Mouse hippocampal neuronal cell line HT22 and human gastric epithelial cell line GES-1 obtained from Procell (Wuhan, China). Rat adrenal medullary chromaffin tumor cell line PC-12 (high differentiation), human fibrosarcoma cell line HT1080, human liver adenocarcinoma cell line SK-Hep-1 and human renal epithelial cell line 293T were obtained from the National Collection of Authenticated Cell Cultures (Shanghai, China). All cell lines were cultured in high glucose DMEM supplemented with 1% penicillin/streptomycin solution and 10% FBS in an incubator (37°C, CO2 5%). Unless otherwise indicated, the final molar concentrations of myriocin, DHS, erastin and glutamate used for experiments were 0.5 μM, 1 μM, 1 μM and 15mM, respectively. When analyzing the cytoprotective effects of myriocin, cells were pre-treated with or without myriocin for 36h before incubated with or without erastin or glutamate for 24h.

### Method details

#### Cell viability assay

Cells were seeded and treated in 96-well plates. After treatment, cell viability was determined by Cell Counting Kit-8 (APExBIO, K1018) according to the manufacture protocol. The absorbance was measured by a microplate reader (Bio-Rad iMark Microplate Reader, USA) at 450 nm.

#### Flow cytometry analysis

After indicated treatment, cells were harvested and stained with Annexin V-FITC/PI (Beyotime, C1062L) or BODIPY 581/591 C11 (Thermo Fisher, D3861) according to the manufacturer’s instructions. Fluorescence signals from about 10000 cells in each sample were recorded by FACS Caliber flow cytometer (Becton Dickinson, San Jose, CA, USA), and data were analyzed using FlowJo_V10 software.

#### Measurement of intracellular ROS

2’,7’-Dichlorofluorescin diacetate (DCFH-DA) fluorescent probe (Sigma-Aldrich, D6883) was used to detect intracellular ROS. Briefly, after indicated treatment, HT22 cells were loaded with 1 μM DCFH-DA (diluted in HBSS buffer) for 30 min at 37°C in the dark. Cells were washed twice with HBSS and monitored by an inverted fluorescence microscope (Leica DMi 8, Leica Microsystems, Germany).

#### Mitochondrial staining

After indicated treatment, HT22 cells were loaded with 50 nM Mito Tracker (diluted in HBSS buffer) for 25 min at 37°C in the dark. Nuclei were stained with DAPI (Beyotime, C1002). Cells were washed twice with HBSS and monitored by an inverted fluorescence microscope (Leica DMi 8, Leica Microsystems, Germany).

#### Western blotting assay

Cells were lysed in Laemmli buffer (0.125 M Tris-HCl, pH 6.8, 2% SDS, 5% β-Mercaptoethanol, 10% glycerine, 0.002% bromophenol blue and 1% protease inhibitor cocktail (APExBIO, K1007)). The lysates were separated on 10% or 12% (wt/vol) SDS-polyacrylamide gels. After SDS/PAGE, proteins were transferred to PVDF membrane for Western blotting in transfer buffer with 10% (vol/vol) methanol at 4°C followed by standard Western-blotting protocols. β-actin or α-Tubulin was used for normalizing the protein load. All blots were repeated at least three times. Comparisons of relative levels of specific antigens were done by quantitative densitometry using ImageJ Software (version 1.48). Antibodies: HIF1α (Cell Signaling Technology, 36169), BNIP3 (Cell Signaling Technology, 3769), Ubiquitin (Cell Signaling Technology, 3936), P70S6K (Cell Signaling Technology, 2708), p-P70S6K (Cell Signaling Technology, 9234), PDK1 (Abcam, ab207450), mTOR (Abcam, ab134903), p-mTOR (Abcam, ab109268), AKT1/2/3 (Abcam, ab179463), p-AKT1/2/3 (Abcam, ab192623), LC3II (Zen BioScience, 306019), β-actin (Zen BioScience, 700068), α-Tubulin (Beyotime, AF0001), peroxidase-conjugated anti-rabbit (Beyotime, A0216) and peroxidase-conjugated anti-mouse antibodies (Beyotime, A0216).

#### Quantitative real-time PCR assay (qPCR)

Total RNA was extracted using Trizol reagent (Invitrogen, 15596026). 1 μg of RNA per sample was reverse-transcribed to cDNA using PrimeScript RT reagent Kit (Takara, RR047A) according to the manufacturer’s specification. Resulting cDNA was analyzed using 2× SYBR Green qPCR Master Mix (APExBIO, K1070) and CFX96 Touch real-time quantitative PCR instrument (Bio-Rad, USA). Gene expression was determined by comparative ΔΔCT method and normalized to the housekeeping gene *Act1*. Primer sequences are listed in Table S2.

#### Glutathione analysis

Glutathione (GSH and GSSG) was detected by GSH and GSSG Assay kit (Beyotime, S0053) according to manufacturer’s protocol. Briefly, to monitor total glutathione (GSSG + GSH), the GSSG in samples was reduced to GSH by glutathione reductase before all GSH was oxidized by the chromogenic substrate of 5,5′-dithiobis-2-nitro- benzoic acid (DTNB) to form the yellow derivative 5′-thio-2-nitrobenzoic acid (TNB). To monitor GSSG along, the endogenous GSH was removed by GSH scavenger before DTNB oxidization. The content of GSH can be calculated by deducting the content of GSSG from total glutathione. The light absorption value of TNB was determined at 412 nm with a microplate reader (Multiskan GO, Thermo Fisher Scientific, USA). Glutathione levels were normalized to protein concentration and expressed as nmol per mg protein.

#### Analysis of glucose metabolites

Cells were seeded and treated with or without myriocin in 6-well plates before being washed in cold PBS and collected by centrifugation. The levels of glucose, pyruvate, lactate, and citrate were detected by colorimetry methods using respective assay kits (Solarbio, BC2500, BC2200, BC2230 and BC2150) according to manufacturer’s instructions.

#### Measurement of the intracellular labile iron pool (LIP)

The intracellular LIP was quantified by monitoring the recovering of calcein fluorescence induced by iron chelators after the calcein fluorescence was quenched by intracellular labile iron (Epsztejn et al., 1997; Liu et al., 2021). Briefly, after indicated treatment, HT22 cells were loaded with 0.25 μM calcein-AM (Sigma-Aldrich, C1359) for 30 min at 37°C. Cells were washed twice with PBS and incubated with 200 μM membrane-permeable iron chelator pyridoxal isonicotinoyl hydrazone (PIH) (Sigma-Aldrich, 528110). Fluorescence of calcein was recorded (excitation: 488nm, emission: 518nm) before (baseline) and 30 min after PIH addition with a Varioskan Flash multimode reader (Thermo Fisher Scientific, USA). The increased fluorescence intensity was normalized for cell number.

#### Immunofluorescence

Cells were washed in PBS, fixed with 4% (vol/vol) paraformaldehyde, permeabilized with 0.3% (vol/vol) Triton X-100, blocked in 5% (vol/vol) bovine serum albumin, and incubated with antibodies HIF1α (Cell Signaling Technology, 36169) using standard protocols. Immunofluorescence was examined and photographed using an inverted fluorescence microscope (Leica DMi 8, Leica Microsystems, Germany).

#### Short hairpin RNA (shRNA) transfections

shHif1α (shHif1α-1: 5′-CCGGTTATGCACTTTGTCGCTATTAATTCAAGAGATTAATAGCGACAAAGTGCATATTTTTTG-3′ and shHif1α-2: 5′-CCGGTTGGATAGCGATATGGTCAATGTTCAAGAGACATTGACCATATCGCTATCCATTTTTTG-3′) or a non-targeting shRNA (Sigma-Aldrich, SHC002) was cloned into pLKO.1-puro empty vectors (Sigma-Aldrich, SHC001). The lentiviral vector was co-transfected into 293T cells with packaging vectors (psPAX2 and pMD2.G) using Lipo6000 (Beyotime, C0526) and cultivated in OptiMEM medium (Gibco, 31985070) for 6h. Cells were further cultured in DMEM complete medium for two days. Then the medium containing lentiviruses was harvested, filtered and used to transfected cells. Transfected cells were selected by 2 μg/ml puromycin. shRNA efficiency was determined by qPCR and western blotting.

#### Co-immunoprecipitation

Proteins were extracted from precleared total cell lysates prepared in RIPA lysis buffer (20 mM Tris-HCl, pH 7.5, 150 mM NaCl, 1% Triton X-100, 0.25% deoxycholic acid, 1 mM EDTA, 1 mM PMSF and 1% protease inhibitor cocktail (APExBIO, K1007)) by incubation with 50 μL of protein A/G agarose beads (Beyotime, P2012) preloaded with 2 μg of Anti-HIF1α antibody (Cell Signaling Technology, 36169). The beads were washed four times with RIPA lysis buffer. Immunoprecipitated proteins were released from the beads by boiling in 2 × Laemmli buffer for 5 min and the supernatant was collected by centrifugation. The proteins in supernatants were separated by SDS-PAGE and analyzed by Western blotting.

#### RNA sequencing analysis (RNA-Seq)

For transcriptome analysis, HT22 cells were treated with or without 0.5 μM myriocin for 36 h. Total RNA was extracted using Trizol reagent according to the manufacturer’s instructions and genomic DNA was removed using DNase I (TaKara, D2215). RNA-seq transcriptome library was prepared with TruSeqTM RNA sample preparation Kit from Illumina (San Diego, RS-122-2002) using 1μg of total RNA for each sample. The synthesized cDNA was subjected to end-repair, phosphorylation, and ‘A’ base addition according to Illumina’s library construction protocol. After quantified by TBS380, paired-end RNA-seq sequencing library was sequenced with the Illumina HiSeq xten. The raw paired end reads were trimmed and quality controlled by SeqPrep (https://github.com/jstjohn/SeqPrep) and Sickle (https://github.com/najoshi/sickle) with default parameters. Then clean reads were aligned to reference genome (Mus_musculus.GRCm38.dna.primary_assembly.fa, version 96.38) with orientation mode using HISAT2 (http://ccb.jhu.edu/software/hisat2/index.shtml, version 2.1.0) (Kim et al., 2019). R statistical package software EdgeR (http://www.bioconductor.org/packages/2.12/bioc/html/edgeR.html, version 2.12) (Robinson et al., 2010) was utilized for differential expression analysis with a filter condition of raw p value < 0.05, and drawing package software ggplot2 (Ginestet, 2011) was utilized for making bar graph, bubble chart, circular dendrogram and heatmap. Functional-enrichment analysis including gene ontology term and KEGG (Kyoto encyclopedia of genes and genomes) was performed by KOBAS 3.0 (http://kobas.cbi.pku.edu.cn/home.do) (Xie et al., 2011).

### Quantification and statistical analysis

The comparisons between two groups were performed by the Student’s t tests (unpaired and two-tailed). Statistical analysis was executed by GraphPad Prism 5.0 software. The number of replicates (n value) is indicated for each figure, and results were presented as average ±SD The p value < 0.05 was considered to reflect a statistically significant difference. ∗p < 0.05, ∗∗p < 0.01, ∗∗∗p < 0.001, ns ＞ 0.05.

## Data Availability

• ·RNA-seq data generated for this article have been deposited at NCBI’s Sequence Read Archive (SRA) and are publicly available as of the date of publication. Accession numbers are listed in the key resources table. • ·This paper does not report original code. • ·Any additional information is available from the lead contact upon reasonable request.

## Supplemental information

Document S1. Figures S1–S8 and Table S2

Table S1. Transcriptome profiles of HT22 cells treated with myriocin, related to Figure 3

## References

[bib1] Albrecht P., Lewerenz J., Dittmer S., Noack R., Maher P., Methner A. (2010). Mechanisms of oxidative glutamate toxicity: the glutamate/cystine antiporter system xc- as a neuroprotective drug target. CNS Neurol. Disord. Drug Targets.

[bib2] Alim I., Caulfield J.T., Chen Y., Swarup V., Geschwind D.H., Ivanova E., Seravalli J., Ai Y., Sansing L.H., Ste Marie E.J. (2019). Selenium drives a transcriptional adaptive program to block ferroptosis and treat stroke. Cell.

[bib3] Bao W.D., Pang P., Zhou X.T., Hu F., Xiong W., Chen K., Wang J., Wang F., Xie D., Hu Y.Z. (2021). Loss of ferroportin induces memory impairment by promoting ferroptosis in Alzheimer's disease. Cell Death Differ..

[bib4] Befani C.D., Vlachostergios P.J., Hatzidaki E., Patrikidou A., Bonanou S., Simos G., Papandreou C.N., Liakos P. (2012). Bortezomib represses HIF-1alpha protein expression and nuclear accumulation by inhibiting both PI3K/Akt/TOR and MAPK pathways in prostate cancer cells. J. Mol. Med. (Berl.).

[bib5] Bekhite M., Gonzalez-Delgado A., Hubner S., Haxhikadrija P., Kretzschmar T., Muller T., Wu J.M.F., Bekfani T., Franz M., Wartenberg M. (2021). The role of ceramide accumulation in human induced pluripotent stem cell-derived cardiomyocytes on mitochondrial oxidative stress and mitophagy. Free Radic. Biol. Med..

[bib6] Boada-Romero E., Martinez J., Heckmann B.L., Green D.R. (2020). The clearance of dead cells by efferocytosis. Nat. Rev. Mol. Cell Biol..

[bib7] Bueno D.C., Canto R.F.S., de Souza V., Andreguetti R.R., Barbosa F.A.R., Naime A.A., Dey P.N., Wullner V., Lopes M.W., Braga A.L. (2020). New probucol analogues inhibit ferroptosis, improve mitochondrial parameters, and induce glutathione peroxidase in HT22 cells. Mol. Neurobiol..

[bib8] Chen J.K., Lane W.S., Schreiber S.L. (1999). The identification of myriocin-binding proteins. Chem. Biol..

[bib9] Chen K., Lin G., Haelterman N.A., Ho T.S., Li T., Li Z., Duraine L., Graham B.H., Jaiswal M., Yamamoto S. (2016). Loss of Frataxin induces iron toxicity, sphingolipid synthesis, and Pdk1/Mef2 activation, leading to neurodegeneration. Elife.

[bib10] Chu J., Liu C.X., Song R., Li Q.L. (2020). Ferrostatin-1 protects HT-22 cells from oxidative toxicity. Neural Regen. Res..

[bib11] Cutler R.G., Thompson K.W., Camandola S., Mack K.T., Mattson M.P. (2014). Sphingolipid metabolism regulates development and lifespan in Caenorhabditis elegans. Mech. Ageing Dev..

[bib12] Dasgupta S., Ray S.K. (2019). Ceramide and sphingosine regulation of myelinogenesis: targeting serine palmitoyltransferase using microRNA in multiple sclerosis. Int. J. Mol. Sci..

[bib13] Di Tano M., Raucci F., Vernieri C., Caffa I., Buono R., Fanti M., Brandhorst S., Curigliano G., Nencioni A., de Braud F., Longo V.D. (2020). Synergistic effect of fasting-mimicking diet and vitamin C against KRAS mutated cancers. Nat. Commun..

[bib14] Dixon S.J., Patel D.N., Welsch M., Skouta R., Lee E.D., Hayano M., Thomas A.G., Gleason C.E., Tatonetti N.P., Slusher B.S., Stockwell B.R. (2014). Pharmacological inhibition of cystine-glutamate exchange induces endoplasmic reticulum stress and ferroptosis. Elife.

[bib15] Doll S., Freitas F.P., Shah R., Aldrovandi M., da Silva M.C., Ingold I., Goya Grocin A., Xavier da Silva T.N., Panzilius E., Scheel C.H. (2019). FSP1 is a glutathione-independent ferroptosis suppressor. Nature.

[bib16] Epsztejn S., Kakhlon O., Glickstein H., Breuer W., Cabantchik I. (1997). Fluorescence analysis of the labile iron pool of mammalian cells. Anal. Biochem..

[bib17] Geekiyanage H., Upadhye A., Chan C. (2013). Inhibition of serine palmitoyltransferase reduces Abeta and tau hyperphosphorylation in a murine model: a safe therapeutic strategy for Alzheimer's disease. Neurobiol. Aging.

[bib18] Ginestet C. (2011). ggplot2: Elegant graphics for data analysis. J. Roy. Stat. Soc..

[bib19] Giussani P., Tringali C., Riboni L., Viani P., Venerando B. (2014). Sphingolipids: key regulators of apoptosis and pivotal players in cancer drug resistance. Int. J. Mol. Sci..

[bib20] Hepowit N.L., Macedo J.K.A., Young L.E.A., Liu K., Sun R.C., MacGurn J.A., Dickson R.C. (2021). Enhancing lifespan of budding yeast by pharmacological lowering of amino acid pools. Aging (Albany NY).

[bib21] Herrera F., Martin V., Garcia-Santos G., Rodriguez-Blanco J., Antolin I., Rodriguez C. (2007). Melatonin prevents glutamate-induced oxytosis in the HT22 mouse hippocampal cell line through an antioxidant effect specifically targeting mitochondria. J. Neurochem..

[bib22] Hu C.L., Nydes M., Shanley K.L., Morales Pantoja I.E., Howard T.A., Bizzozero O.A. (2019). Reduced expression of the ferroptosis inhibitor glutathione peroxidase-4 in multiple sclerosis and experimental autoimmune encephalomyelitis. J. Neurochem..

[bib23] Huang X., Liu J., Dickson R.C. (2012). Down-regulating sphingolipid synthesis increases yeast lifespan. PLoS Genet..

[bib24] Jiang T., Cheng H., Su J., Wang X., Wang Q., Chu J., Li Q. (2020). Gastrodin protects against glutamate-induced ferroptosis in HT-22 cells through Nrf2/HO-1 signaling pathway. Toxicol. Vitro.

[bib25] Kapralov A.A., Yang Q., Dar H.H., Tyurina Y.Y., Anthonymuthu T.S., Kim R., St Croix C.M., Mikulska-Ruminska K., Liu B., Shrivastava I.H. (2020). Redox lipid reprogramming commands susceptibility of macrophages and microglia to ferroptotic death. Nat. Chem. Biol..

[bib26] Kim D., Paggi J.M., Park C., Bennett C., Salzberg S.L. (2019). Graph-based genome alignment and genotyping with HISAT2 and HISAT-genotype. Nat. Biotechnol..

[bib27] Kim J.W., Tchernyshyov I., Semenza G.L., Dang C.V. (2006). HIF-1-mediated expression of pyruvate dehydrogenase kinase: a metabolic switch required for cellular adaptation to hypoxia. Cell Metab..

[bib28] Lin G., Lee P.T., Chen K., Mao D., Tan K.L., Zuo Z., Lin W.W., Wang L., Bellen H.J. (2018). Phospholipase PLA2G6, a Parkinsonism-associated gene, affects Vps26 and Vps35, retromer function, and ceramide levels, similar to alpha-Synuclein gain. Cell Metab..

[bib29] Lin G., Wang L., Marcogliese P.C., Bellen H.J. (2019). Sphingolipids in the pathogenesis of Parkinson's disease and parkinsonism. Trends Endocrinol. Metab..

[bib30] Liu B., Wang W., Shah A., Yu M., Liu Y., He L., Dang J., Yang L., Yan M., Ying Y. (2021). Sodium iodate induces ferroptosis in human retinal pigment epithelium ARPE-19 cells. Cell Death Dis..

[bib31] Liu J., Huang X., Withers B.R., Blalock E., Liu K., Dickson R.C. (2013). Reducing sphingolipid synthesis orchestrates global changes to extend yeast lifespan. Aging Cell.

[bib32] Liu J., Yang M., Kang R., Klionsky D.J., Tang D. (2019). Autophagic degradation of the circadian clock regulator promotes ferroptosis. Autophagy.

[bib33] Lu Z., Imlay J.A. (2019). A conserved motif liganding the [4Fe-4S] cluster in [4Fe-4S] fumarases prevents irreversible inactivation of the enzyme during hydrogen peroxide stress. Redox Biol..

[bib34] Maher P., Currais A., Schubert D. (2020). Using the oxytosis/ferroptosis pathway to understand and treat age-associated neurodegenerative diseases. Cell Chem. Biol..

[bib35] Maher P., Davis J.B. (1996). The role of monoamine metabolism in oxidative glutamate toxicity. J. Neurosci..

[bib36] Mahoney-Sanchez L., Bouchaoui H., Ayton S., Devos D., Duce J.A., Devedjian J.C. (2021). Ferroptosis and its potential role in the physiopathology of Parkinson's Disease. Prog. Neurobiol..

[bib37] Masoud G.N., Li W. (2015). HIF-1alpha pathway: role, regulation and intervention for cancer therapy. Acta Pharm. Sin. B.

[bib38] Mi C., Ma J., Wang K.S., Zuo H.X., Wang Z., Li M.Y., Piao L.X., Xu G.H., Li X., Quan Z.S., Jin X. (2017). Imperatorin suppresses proliferation and angiogenesis of human colon cancer cell by targeting HIF-1alpha via the mTOR/p70S6K/4E-BP1 and MAPK pathways. J. Ethnopharmacol..

[bib39] Mi Y., Gao X., Xu H., Cui Y., Zhang Y., Gou X. (2019). The emerging roles of ferroptosis in Huntington's disease. NeuroMolecular Med..

[bib40] Miyake Y., Kozutsumi Y., Nakamura S., Fujita T., Kawasaki T. (1995). Serine palmitoyltransferase is the primary target of a sphingosine-like immunosuppressant, ISP-1/myriocin. Biochem. Biophys. Res. Commun..

[bib41] Murphy T.H., Miyamoto M., Sastre A., Schnaar R.L., Coyle J.T. (1989). Glutamate toxicity in a neuronal cell line involves inhibition of cystine transport leading to oxidative stress. Neuron.

[bib42] Neitemeier S., Jelinek A., Laino V., Hoffmann L., Eisenbach I., Eying R., Ganjam G.K., Dolga A.M., Oppermann S., Culmsee C. (2017). BID links ferroptosis to mitochondrial cell death pathways. Redox Biol..

[bib43] Novgorodov S.A., Gudz T.I. (2011). Ceramide and mitochondria in ischemic brain injury. Int J. Biochem Mol. Biol..

[bib44] Paltoglou S., Roberts B.J. (2007). HIF-1alpha and EPAS ubiquitination mediated by the VHL tumour suppressor involves flexibility in the ubiquitination mechanism, similar to other RING E3 ligases. Oncogene.

[bib45] Petit C.S., Lee J.J., Boland S., Swarup S., Christiano R., Lai Z.W., Mejhert N., Elliott S.D., McFall D., Haque S. (2020). Inhibition of sphingolipid synthesis improves outcomes and survival in GARP mutant wobbler mice, a model of motor neuron degeneration. Proc. Natl. Acad. Sci. USA..

[bib46] Poloznikov A.A., Nersisyan S.A., Hushpulian D.M., Kazakov E.H., Tonevitsky A.G., Kazakov S.V., Vechorko V.I., Nikulin S.V., Makarova J.A., Gazaryan I.G. (2020). HIF Prolyl Hydroxylase inhibitors for COVID-19 treatment: Pros and Cons. Front. Pharmacol..

[bib47] Robinson M.D., McCarthy D.J., Smyth G.K. (2010). edgeR: a Bioconductor package for differential expression analysis of digital gene expression data. Bioinformatics.

[bib48] Samarani M., Loberto N., Solda G., Straniero L., Asselta R., Duga S., Lunghi G., Zucca F.A., Mauri L., Ciampa M.G. (2018). A lysosome-plasma membrane-sphingolipid axis linking lysosomal storage to cell growth arrest. FASEB J.

[bib49] Seo Y.J., Alexander S., Hahm B. (2011). Does cytokine signaling link sphingolipid metabolism to host defense and immunity against virus infections?. Cytokine Growth Factor Rev..

[bib50] Tang D., Kang R., Berghe T.V., Vandenabeele P., Kroemer G. (2019). The molecular machinery of regulated cell death. Cell Res..

[bib51] Testai F.D., Kilkus J.P., Berdyshev E., Gorshkova I., Natarajan V., Dawson G. (2014). Multiple sphingolipid abnormalities following cerebral microendothelial hypoxia. J. Neurochem..

[bib52] Torti S.V., Torti F.M. (2013). Iron and cancer: more ore to be mined. Nat. Rev. Cancer.

[bib53] Wadsworth J.M., Clarke D.J., McMahon S.A., Lowther J.P., Beattie A.E., Langridge-Smith P.R., Broughton H.B., Dunn T.M., Naismith J.H., Campopiano D.J. (2013). The chemical basis of serine palmitoyltransferase inhibition by myriocin. J. Am. Chem. Soc..

[bib54] Wang L., Liu Y., Du T., Yang H., Lei L., Guo M., Ding H.F., Zhang J., Wang H., Chen X., Yan C. (2020). ATF3 promotes erastin-induced ferroptosis by suppressing system Xc(.). Cell Death Differ..

[bib55] Xie C., Mao X., Huang J., Ding Y., Wu J., Dong S., Kong L., Gao G., Li C.Y., Wei L. (2011). Kobas 2.0: a web server for annotation and identification of enriched pathways and diseases. Nucleic Acids Res..

[bib56] Yang M., Chen P., Liu J., Zhu S., Kroemer G., Klionsky D.J., Lotze M.T., Zeh H.J., Kang R., Tang D. (2019). Clockophagy is a novel selective autophagy process favoring ferroptosis. Sci. Adv..

[bib57] Zhang H., Bosch-Marce M., Shimoda L.A., Tan Y.S., Baek J.H., Wesley J.B., Gonzalez F.J., Semenza G.L. (2008). Mitochondrial autophagy is an HIF-1-dependent adaptive metabolic response to hypoxia. J. Biol. Chem..

[bib58] Zhang Y., Fan B.Y., Pang Y.L., Shen W.Y., Wang X., Zhao C.X., Li W.X., Liu C., Kong X.H., Ning G.Z. (2020). Neuroprotective effect of deferoxamine on erastininduced ferroptosis in primary cortical neurons. Neural Regen. Res..

