# Supplementary material for: Pharmacological inhibition of sphingolipid synthesis reduces ferroptosis by stimulating the HIF-1 pathway
Source: iScience. 2022 Jun 6;25(7):104533. doi: 10.1016/j.isci.2022.104533 (PMC9240796; doi:10.1016/j.isci.2022.104533)
Supplement: Document S1. Figures S1–S8 and Table S2 [file mmc1.pdf]

iScience, Volume *25*

Supplemental information

Pharmacological inhibition of sphingolipid synthesis reduces ferroptosis by stimulating the HIF-1 pathway

Yang Liu, Libo He, Binghua Liu, Yuling Ying, Junling Xu, Meng Yu, Jinye Dang, and Ke Liu

Supplemental Figures

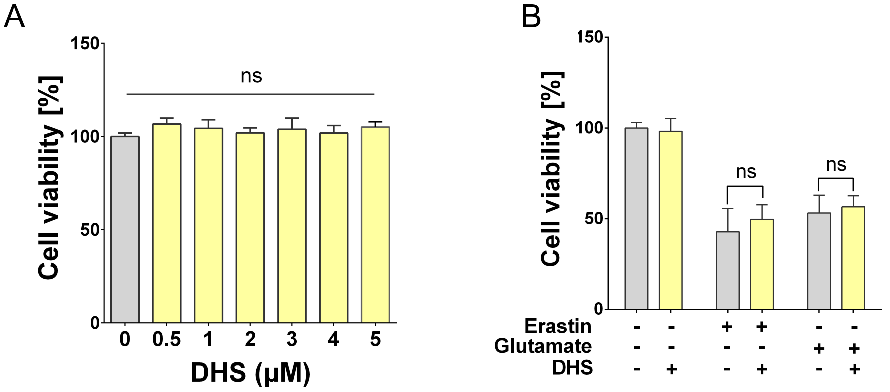

Figure S1

DHS alone has no impact on cell viability (Related to Figure 1). **A** Cell viability analysis of cells treated with DHS for 36h. **B** Cell viability analysis of cells pre-treated with DHS (1 µM) for 36h before incubating with erastin (1 µM) or glutamate (15 mM) for 24h. Error bars represent the mean ± SD (n = 3, ns, no significant).

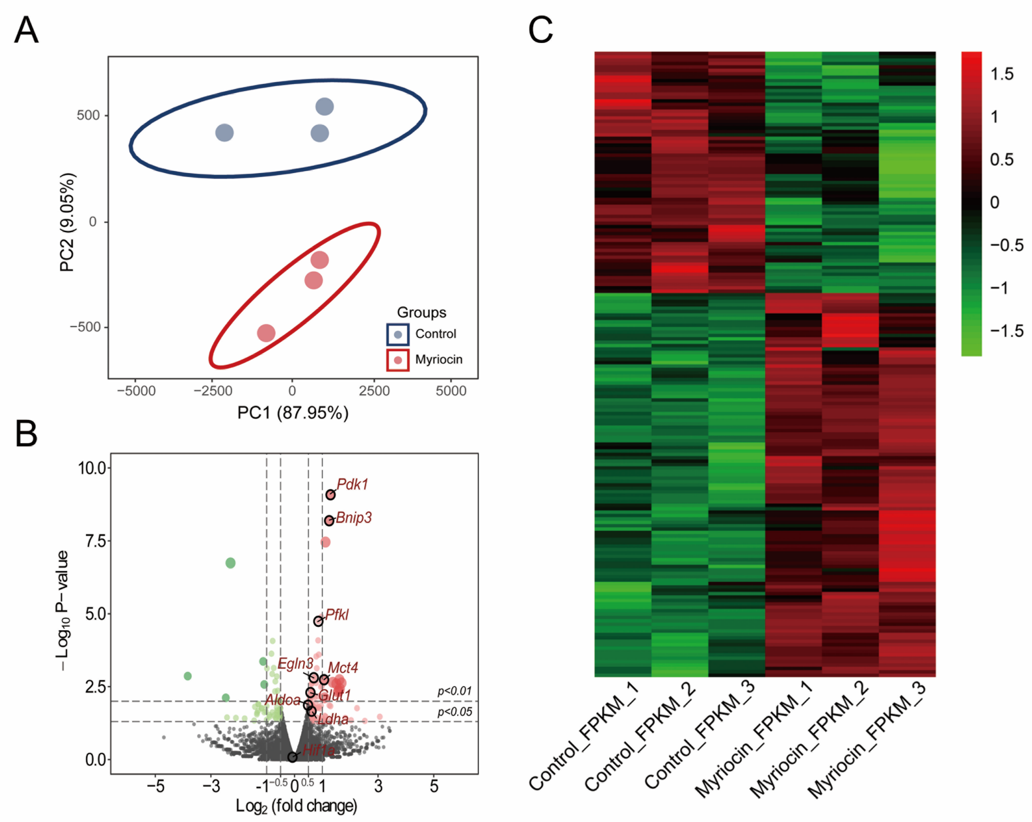

Figure S2

RNA sequencing analysis of HT22 cells treated with or without myriocin (0.5 µM) for 36 h (Related to Figure 3). **A** Principal component analysis (PCA) score plot of gene expression profiles. **B** Volcano plots of gene expression profiles. The black circle presents the genes verified in Figure 4A. **C** Heatmap of expression (Log2FC) of all differentially expressed genes (p-value < 0.05).

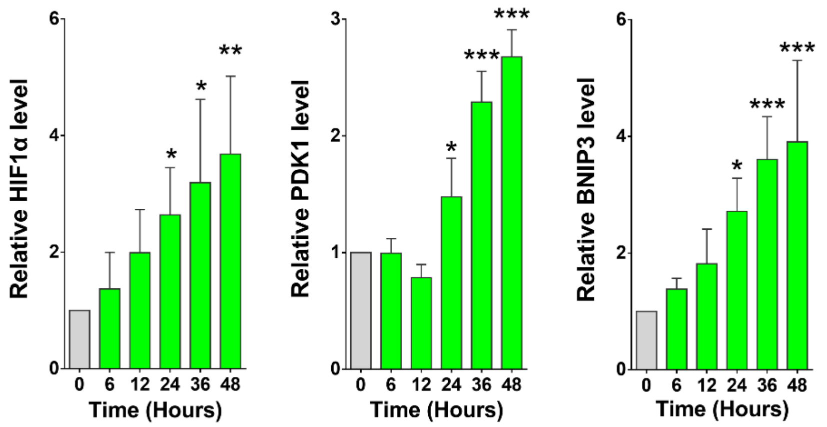

Figure S3

Myriocin increases protein levels of HIF1α, PDK1 and BNIP3 (Related to Figure 4). Western blots in Figure 4C were quantified using ImageJ software. Data were normalized to relevant untreated control. Error bars represent the mean ± SD (n = 3, * $p < 0.05$, ** $p < 0.01$, *** $p < 0.001$, ns, no significant).

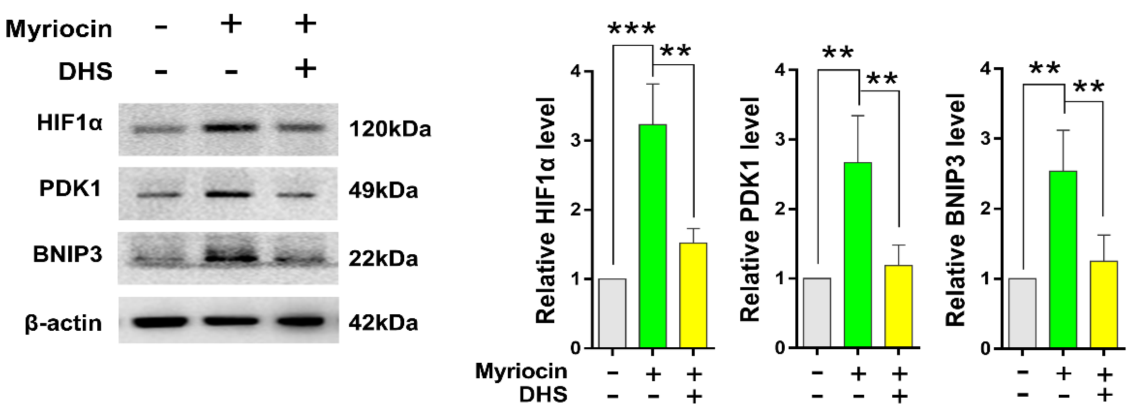

Figure S4

Western blotting analysis of HIF1α, PDK1 and BNIP3 protein in HT22 cells treated with or without myriocin (0.5 μM) in the presence or absence of DHS (1 μM) (Related to Figure 4). Bar graph shows the quantification of protein expression relative to the relevant untreated control. Error bars represent the mean ± SD (n = 3, * $p < 0.05$, ** $p < 0.01$, *** $p < 0.001$).

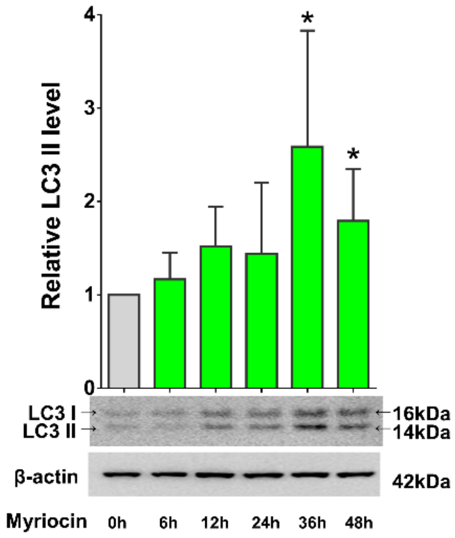

Figure S5

Myriocin increases LC3II in a time dependent manner (Related to Figure 4). HT22 cells were treated with myriocin (0.5 μM) for different time and subjected to Western blotting for LC3II. Bar graph shows the quantification of protein expression relative to the relevant untreated control. Error bars represent the mean ± SD (n = 3, * $p < 0.05$).

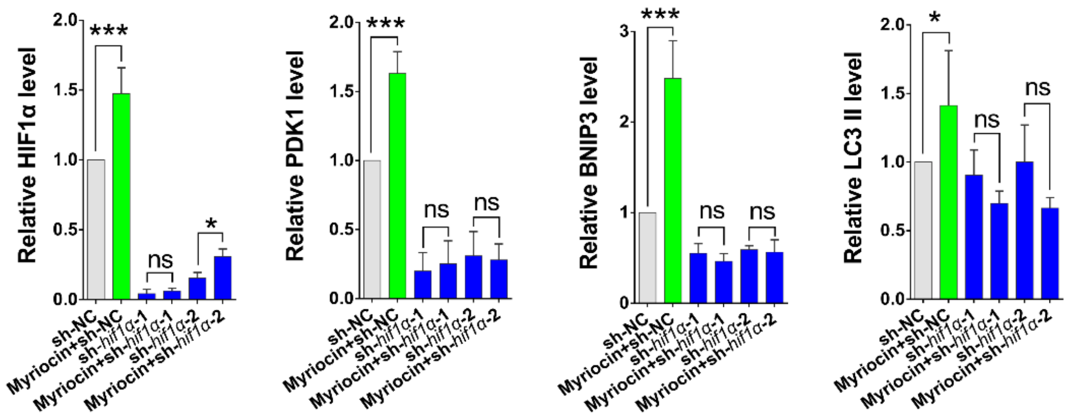

Figure S6

Hif1α knockdown decreases HIF1α, PDK1, BNIP3 and LC3 II (Related to Figure 5). Western blots in Figure 5B were quantified using ImageJ software. Data were normalized to relevant untreated control. Error bars represent the mean ± SD (n = 3, * $p < 0.05$, ** $p < 0.01$, *** $p < 0.001$, ns, no significant).

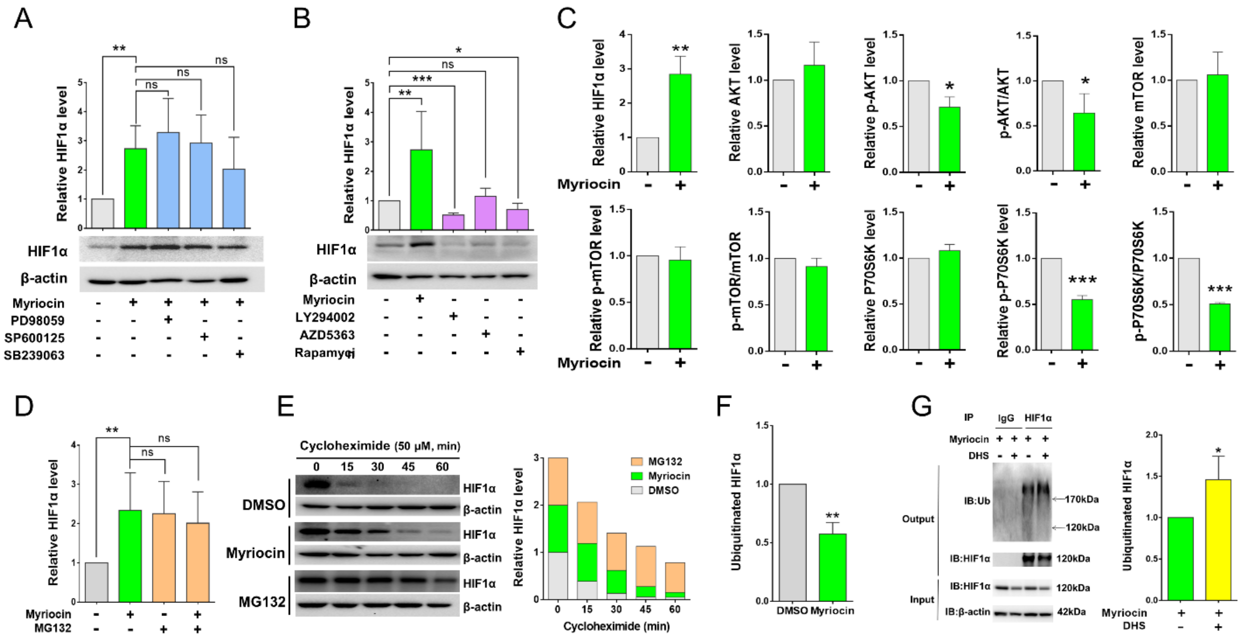

Figure S7

Myriocin activates the HIF-1 pathway independent of MAPK or PI3K pathways (Related to Figure 6). **A** Western blotting analysis of HIF1α protein in HT22 cells treated with myriocin (0.5 μM) in the presence or absence of PD98059 (10 μM), SP600125 (10 μM) or SB239063 (10 μM) for 36h. **B** Western blotting analysis of HIF1α protein in HT22 cells treated with myriocin (0.5 μM), LY294002 (10 μM), AZD5363 (5 μM) or Rapamycin (1 μM) for 36h. **C** The quantification of western blots in Figure 6C. **D** The quantification of western blots in Figure 6D. **E** Western blotting analysis of HIF1α protein in HT22 cells treated with myriocin (0.5 μM, 36h) or MG132 (10 μM, 4h) before incubating with 50μM cycloheximide for indicated time. **F** The quantification of western blots in Figure 6E. **G** Ubiquitin/HIF1α co-immunoprecipitation in HT22 cell extracts. Cells treated with myriocin (0.5 μM) in the presence or absence of DHS (1 μM) for 36h were lysed and immunoprecipitated with anti-HIF1α antibody or normal immunoglobin G (IgG) as negative control. The immunocomplexes were then immunoblotted using anti-ubiquitin or anti-HIF1α antibody (Output). The levels of HIF1α in cell lysates before immunoprecipitation were also monitored (Input). Bar graph shows the quantification of protein expression relative to the relevant untreated control. Error bars represent the mean ± SD (n = 3, * $p < 0.05$, ** $p < 0.01$, *** $p < 0.001$, ns, no significant).

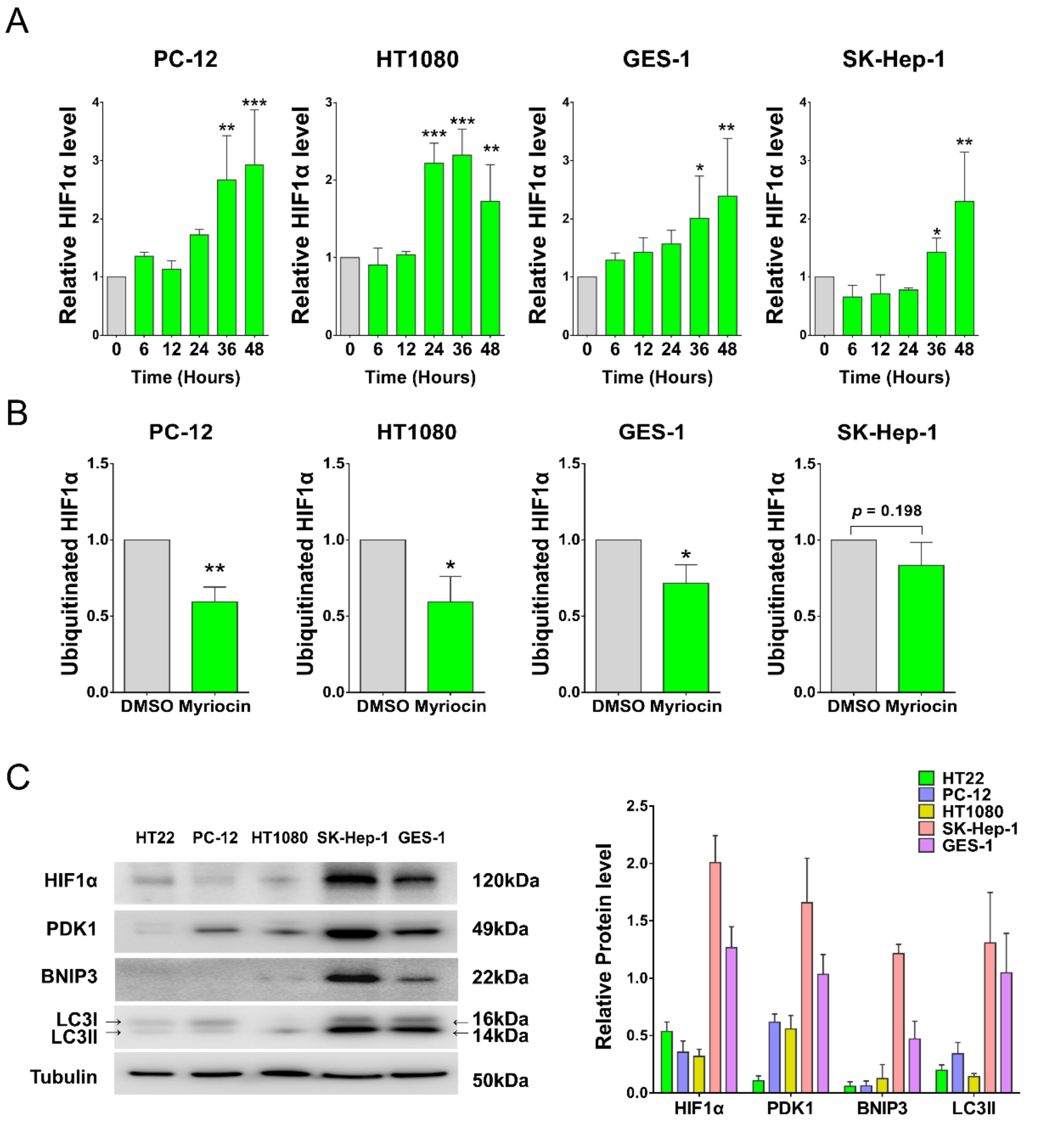

Figure S8

Myriocin stabilizes HIF1α in different cell lines (Related to Figure 7). **A** The quantification of western blots in Figure 7A. **B** The quantification of western blots in Figure 7C. **C** Proteins of interested in HT22, PC-12, HT1080, GES-1 and SK-Hep-1 cells were tested by Western blotting. Bar graph shows the quantification of protein expression relative to the relevant untreated control. Error bars represent the mean ± SD (n = 3, * $p < 0.05$, ** $p < 0.01$, *** $p < 0.001$, ns, no significant).

Table S2. qPCR primer sequences (Related to STAR Methods)

Genes	Forward primer (5’-3’)	Reverse primer (5’-3’)
Hif1α	AAGTCAGCAACGTGGAAGGT	ATCAGCACCAAGCACGTCAT
Pdk1	ACGGGACAGATGCGGTTATC	GCGTCCCATGTGCGTTTAAG
Bnip3	AGGCGTCTGACAACTTCCAC	CCAAGGACCATGCTAGCTCT
Egln3	AATGGTGATGGCCGCTGTAT	GCATAGGAGGGCTGGACTTC
Glut1	ACCATCTTGGAGCTGTTCCG	GCCTTCTCGAAGATGCTCGT
Aldoa	AACATACTCCCTCGGCCCT	TCAGTAACATACTGGCAGCG
Pfkl	TCCGCACCTACAACATCCAC	GGCTGGGATGACACACATGA
Ldha	ACTCAAGGGCGAGATGATGG	CCAGCTTGGAGTTCGCAGTT
Shc4	GGAAGCAATAAGTCGCCTGTG	ATCAGTGTGAGGCTGCTTGT
Actb	TGTACCCAGGCATTGCTGAC	AACGCAGCTCAGTAACAGTCC
Human-hif1α	CCTATGACCTGCTTGGTGCT	GTCCTGTGGTGACTTGTCCT
Human-Actb	GCTCACCATGGATGATGATATCGC	TAGGAATCCTTCTGACCCATGCC
Rat-hif1α	AATCTGAGGACACGAGCTGC	GCTGCCGAAGTCCAGTGATA
Rat-Actb	CCCGCGAGTACAACCTTCT	ATACCCACCATCACACCCTG
